# The Influence of the Site of Recording and Benchtop and Portable NIRS Equipment on Predicting the Sensory Properties of Iberian Ham

**DOI:** 10.3390/foods15030436

**Published:** 2026-01-24

**Authors:** Isabel Revilla, Ana María Vivar-Quintana, Iván Martínez-Martín, Pedro Hernández-Ramos, Miriam Hernández-Jiménez, Justyna Grabska, Krzysztof B. Beć, Christian W. Huck

**Affiliations:** 1 Area of Food Technology, Polytechnical Superior School of Zamora, Universidad de Salamanca, Avenida Requejo 33, 49022 Zamora, Spain; avivar@usal.es (A.M.V.-Q.); ivanm@usal.es (I.M.-M.); 2 Area of Graphic Expression in Engineering, Polytechnical Superior School of Zamora, Universidad de Salamanca, Avenida Requejo 33, 49022 Zamora, Spain; pedrohde@usal.es; 3 Science and Arts Faculty, Universidad Católica de Avila, Calle Canteros s/n, 05005 Avila, Spain; miriam.hernandez1@ucavila.es; 4 Institute of Analytical Chemistry and Radiochemistry, Leopold-Franzens University of Innsbruck, 6020 Innsbruck, Austria; justyna.grabska@uibk.ac.at (J.G.); krzysztof.bec@uibk.ac.at (K.B.B.); christian.w.huck@uibk.ac.at (C.W.H.)

**Keywords:** near infrared spectroscopy, Iberian ham, fat, lean meat, whole slices, instrumental difference

## Abstract

Iberian ham is a Spanish product that is highly prized for its unique sensory characteristics. The possibility of predicting these characteristics using non-invasive methods is therefore of great interest. In this context, this study compares the performance of two benchtop and four portable devices in addition to the influence of recording factors (lean meat, fat, and whole slices) for the prediction of sensory parameters. A total of 28 descriptors were determined by a trained panel for 60 samples (100% Iberian and Iberian), and the sensory profile was predicted by using a feedforward multilayer perceptron artificial neural network. A significant effect of the breed on the sensory profile and the spectral characteristics was observed, especially in the case of fat. On the other hand, the NIRFlex N-500 (desktop) and MicroPhazir (portable) devices gave the best performances, with 27 parameters predicted for whole slices and fat, respectively (R^2^ > 0.5). In addition, it was possible to predict 25 parameters by using the MicroNIR portable device at all recording sites, which demonstrated that portable devices are suitable for this analysis. The results indicate that the number and type of sensory parameters predicted depend largely on the recording site and that measurements taken in different areas provide complementary information.

## 1. Introduction

Iberian ham is a pork product that is not only highly appreciated by Spanish consumers but is also one of the most recognized Spanish products in the world; it is marketed in Europe, Japan, and the United States [1]. One of the reasons why it is in such high demand is the sensory quality of this product, which depends on several factors, such as genetics, breeding systems, and especially the feed provided [2], with 100% acorn-fed Iberian ham being the most prized for its aroma, texture, and flavor [3]. Iberian ham is characterized by the intense red color of its lean meat with a large amount of white intramuscular fat (marbling). In addition, its low fiber content, high juiciness, and soft, fluid fat as texture attributes, together with a high intensity of flavor owing to its cured taste with hints of sweetness, are the main characteristics that influence the acceptability of cured ham [4]. There is, therefore, a complete set of sensory attributes that sensory panels analyze when performing quantitative descriptive analysis (QDA), the most widely used descriptive method, to evaluate quality and to differentiate cured Iberian hams [5,6,7,8,9]. However, the implementation of QDA involves the selection, training, and qualification of evaluators in accordance with recognized standards [10,11,12], and also the validation of the panel to ensure its correct functioning over time [6,13]. Consequently, this method is tedious and time-consuming, in addition to requiring the destruction of the sample and a panel of assessors undergoing continuous training. However, the data obtained are not only reliable but also provide a significant amount of complementary information, which is easy to interpret [14].

As a result of all this, it is of great interest to replace the sensory analysis by non-destructive techniques such as near-infrared spectroscopy, which has advantages such as being quick and non-contaminant, and also offers the possibility of implementation on-line/in-line or at-line [15]. NIR spectroscopy has been used to predict sensory properties in different matrices, as reviewed by Chapman et al. [16]. With regard to meat and meat products, much more attention has been paid to fresh meat than to meat products [17], although some recent studies point to the feasibility of predicting the sensory properties of dry-cured products [18,19,20], including the texture and color or defects of cured ham [21,22] or even the complete QDA profile [8]. Portable devices are available that differ in technical solutions regarding the light source, the detectors, and the wavelength selectors. The light source of handheld NIR spectrometers is therefore based on tungsten-halogen lamps in analogy to benchtop instruments, but light-emitting diodes (LEDs) are also used (SCiO, Consumer Physics, Tel Aviv, Israel) due to their robustness, low price, and low power consumption. As far as detectors are concerned, array detectors (MicroNIR1700, VIAVI Solutions Inc., Milpitas, CA, USA) and single-detector devices (Enterprise Sensor NIR, TellSpec, Toronto, ON, Canada) can be found in portable NIR equipment. Indium gallium arsenide (InGaAs) is the preferred material for manufacturing detectors and has replaced lead salt detectors PbSe and PbS (Foss NIRSystem 5000, Foss NIRSystems, Silver Spring, MD, USA), although complementary metal–oxide–semiconductor (CMOS) technology has been gaining ground steadily, as this requires less power consumption (SCiO Consumer Physics, Tel Aviv, Israel). As for wavelength selectors, the equipment can currently be divided into the following: Linear-variable filter instruments with array detectors (trinamiX GmbH, Ludwigshafen, Germany; VIAVI Solutions Inc., CA, USA), MEMS-based FT-NIR instruments (Si-Ware Systems, Cairo, Egypt; Hamamatsu Photonics, Hamamatsu City, Japan), MEMS Hadamard masks (microPHAZIR, Thermo Fisher Scientific, Waltham, MA, USA), micro-mirror devices (DMD™) (Texas Instruments, Dallas, TX, USA; Innospectra Corp., Xinzhu, Taiwan, China), MEMS Fabry–Perot interferometers (Nirone Sensor S, Spectral Engines, Helsinki, Finland), NIR grating micro-spectrometers, (Insion GmbH, Obersulm, Germany; OrO Photonics, Xinzhu, Taiwan, China), and NIR scanners with 16 solar cell detectors (Senorics GmbH, Dresden, Germany) [23,24].

This diversity of technological solutions leads to different operational wavelength regions, spectral resolutions, and signal-to-noise ratios [23], which may affect the analytical performance or applicability to a specific matrix. However, in recent years, studies have shown that similar results can be obtained with benchtop and handheld devices for both quantitative and qualitative applications [25,26,27]. The performance of the handheld devices against the benchtop instruments should therefore be tested for each application, as they can be considered a reference for evaluating the analytical performance of portable spectrometers.

In this context, the objective of this study was to investigate the feasibility of predicting the sensory parameters of Iberian ham using different benchtop devices—Foss NIRSystem 5000 (Foss NIRSystems, Silver Spring, MD, USA) and BüchiNIRFlex N-500 (Büchi, Flawil, Switzerland)—and portable equipment—microPHAZIR (Thermo Fischer Scientific, USA); MicroNirProEs (VIAVI solutions, USA); SCiO (Consumer Physics, Israel); and Enterprise Sensor NIR (TellSpec, UK)—as they encompass a wide range of technological solutions, including different light sources, wavelength selectors, detectors, resolutions, and spectral intervals. On the other hand, the influence of recording factors (fat, lean meat, or whole slices) in order to determine the most appropriate methodology for determining the sensory profile easily and accurately was also studied. Achieving this objective will allow the use of NIRS handheld devices for sensory analysis to be implemented routinely in the meat sector.

## 2. Materials and Methods

### 2.1. Materials

A total of 60 samples of Iberian ham were selected, with 24 samples from the 100% Iberian acorn-fed category and 36 samples from the Iberian (Iberian x Duroc) acorn-fed category. All animals were raised in the same geographical area according to certificate RD 10/2014 [28], as previously described by Hernández-Jiménez et al. [27]. One hundred grams of 1 mm-thick ham slices (including *Biceps femoris*, *Semimembranosus*, and *Semitendinosus* muscles) were analyzed when taken perpendicularly to the bone at the same depth from the front of the hams.

### 2.2. Sensory Analysis

The sensory analysis of the samples was carried out according to the methodology described previously by Hernández-Ramos et al. [8] using a sensory panel of 10 members who had been trained in quantitative descriptive analysis (QDA) of cured ham. The assessors gave their written consent to participate before the start of the test. Ethical approval was obtained from the Ethics Committee of the University of Salamanca (registration number 1298). Prior to the analysis, the panel received training during six sessions using reference products and dummy samples. At the end of each session, the director provided feedback on performance using statistical descriptors. After the training phase, repeatability and reproducibility were calculated [29] by analyzing the same sample three times in three different sessions in order to evaluate the accuracy of the panel.

A total of 28 parameters were analyzed, including the visual, flavor, and texture properties of the samples (Supplementary Material, Table S1). A structured scoring scale was used, ranging from 1, low intensity, or the presence of the parameter evaluated, to 9, high intensity, or the presence of the parameter evaluated. The 60 samples, tempered to 20 ± 2 °C and coded, were presented and tasted individually during 15 sessions (4 samples per session).

### 2.3. Near-Infrared Spectroscopy Analysis

Prior to recording, the ham packages were tempered for two hours until the samples reached a temperature of 20 ± 2 °C. Four slices were separated from each package and placed on top of each other, facing in the same direction.

All samples were analyzed using two types of benchtop equipment, Foss NIRSystem 5000 (Foss NIRSystems, Silver Spring, MD, USA) and BüchiNIRFlex N-500 (Büchi, Flawil, Switzerland), and four portable devices: MicroNIR 1700 ES (VIAVI, Milpitas, CA, USA); Enterprise Sensor (TellSpec, Toronto, ON, Canada); SCiO Sensor (Consumer Physics, Tel Aviv, Israel); and microPHAZIR (Thermo Fisher Scientific, Waltham, MA, USA). The main features and technical parameters of the devices, in addition to the spectral range, recording interval, and the measurement of references, are described in the Supplementary Material (Table S2).

The NIR spectra were recorded by placing the device window directly on the surface of the portion. The Foss NIRSystem 5000 was equipped with a 5 cm × 5 cm quartz window so that the spectrum area included both lean muscle mass and fat. The spectra were recorded at three different points, and the average of these was considered to be the spectrum for the whole slice. In the case of the BüchiNIRFlex N-500 500 (Büchi, Flawil, Switzerland)microPHAZIR (Thermo Fisher Scientific, Waltham, MA, USA), MicroNIR 1700 ES (VIAVI, Milpitas, CA, USA), Enterprise Sensor (TellSpec, Toronto, ON, Canada), and SCiO (Consumer Physics, Tel Aviv, Israel) devices, six spectra of lean muscle and four spectra of fat were recorded. Subsequently, the mean of all replicates in each area was calculated to obtain a mean spectrum for fat and a mean spectrum for lean meat, and the mean spectrum for the entire slice was calculated as the mean of all spectra. Each sample was recorded by all devices in the same session.

### 2.4. Artificial Neural Network Calibration

The calibration curves for all sensory parameters were calculated by using a feedforward multilayer perceptron (MLP) artificial neural network (ANN), employing a Levenberg–Marquardt backpropagation training algorithm in accordance with the protocol previously described by Hernández-Ramos et al. [8].

The ANN selected consisted of an input layer that had as many neurons as NIR absorbance values. The ANN developed for the Foss NIRSystem 5000, therefore, had 451 neurons; for the BüchiNIRFlex N-500, it had 1501 neurons; for the MicroNIR 1700, it had 125 neurons; for the Enterprise Sensor, it had 256 neurons; and for the SCiO Sensor, it had 331 neurons, and for the microPHAZIR, it had 100 neurons. All ANNs had a hidden layer with a number of neurons ranging from 1 to 15. Finally, the output layer had only one neuron, which gave the predicted value for each parameter. The selected functions were the hyperbolic tangent sigmoid for the hidden layer and the pure linear transfer function for the output layer. For each scenario (device, recording area, and sensory parameter) and number of neurons in the hidden layer, the ANN was trained 100 times to obtain the best architecture using a known seed value as the initial weight and a bias matrix to allow the subsequent reproducibility of the data [30]. The pairs of input (absorbance value) and output (sensory score) data were randomly divided for all ANNs into three sets called the training set, validation set, and test set to represent 70%, 15%, and 15% of the data, respectively, i.e., a total of 42 samples in the training set and 9 samples in the validation and test sets. A total of 672,000 architectures were calculated (28 parameters, 5 devices, 3 recording sites, 100 training times, and between 1 and 15 neurons, plus 28 parameters, 1 device, 1 recording site, 100 training times, and between 1 and 15 neurons), and the best architecture was selected based on the highest R^2^ values and the lowest RMSE values. The RPD (ratio performance deviation) was calculated for the best final ANN architecture in order to assess the model performance. The Deep Learning Toolbox of MatLab (MathWorks^®^) version R2018 was the software used for all tests.

## 3. Results

### 3.1. Sensory Characteristics of the Samples Analyzed

The sensory characteristics or attributes of the samples analyzed by the sensory panel are shown in Table 1. Statistical analysis of the samples showed significant differences between the 100% Iberian and Iberian samples in four out of the six visual appearance parameters and seven out of the eight texture parameters, while significant differences were only observed in one of the parameters included in the flavor profile, rancid aroma. It is worth noting that, as described in the Materials and Methods, both groups of samples were processed in the same factory, following exactly the same protocol, and were cured in the same facilities for the same period of time. Therefore, the significant differences observed were mainly related to breed, as both groups were raised in the same geographical area and under the same management system, the *montanera*, in which the animals feed on grass and acorns during the fattening period.

The purebred Iberian samples were characterized by significantly less marbling, less color homogeneity, and fewer white spots, while color intensity was higher. These results are fully in agreement with those previously published by Hernández-Ramos et al. [8], who analyzed 100% Iberian and Iberian commercial hams. They attributed the increased presence of veining and exudate to the higher intramuscular fat content of the Iberian breed, as also reported by Mayoral et al. [31]. In terms of color, the 100% Iberian breed showed greater color intensity and less chromatic homogeneity, i.e., the presence of several shades in the same slice, which is consistent with the higher L* and a* values and higher myoglobin content of pure Iberian hams described by Fuentes et al. [32]. The samples analyzed were characterized by the presence of white spots, which are tyrosine and phenylalanine crystals that appear as a result of proteolysis when hams are aged for long periods [33]. Previous results have shown that the incidence of tyrosine crystals depends on the salinity, drying level, temperature, and the combined effect [34]. However, the results of both these samples and commercial samples [8] point to a significant influence of the breed on this phenomenon. In this regard, Carrapiso et al. [35] described that significant differences in bitterness, which are also related to the proteolysis process [36], were only related to crossbreeding.

Flavor parameters were not significantly affected by the breed, which is consistent with previous results showing no major significant differences between 100% and 50% Iberian pigs [8,35] attributed to genetic similarity. It should be noted that some attributes, such as saltiness, sourness, rancidity, or an atypical flavor, showed almost identical values, which shows that these parameters were more correlated with processing conditions. On the other hand, the odor intensity, cured aroma, rancidity, flavor intensity, and fat flavor intensity, although they showed no statistically significant differences, were slightly higher in 50% Iberian ham, which is probably due to the higher fat content. It is well-known that lipolysis, fatty acid oxidation, and Maillard reactions constitute the main processes contributing to the generation of the volatile responsible for the odor and flavor of cured ham [37,38,39]. In addition, proteolysis is also involved in the generation of volatile compounds [39] and the formed products formed either contribute directly to the taste [40] or are precursors of odorants [41]. Differences in proteolysis related to crossbreeding could, therefore, also affect the flavor profile.

Finally, texture attributes were affected by genetics in the same way, as previously reported by Hernández-Ramos et al. [8], which demonstrates the clear effect of crossbreeding on the texture of Iberian ham. The 50% Iberian ham samples were significantly less tough and fibrous and had lower chewiness and gumminess values, although these were not statistically significant. These samples also showed significantly higher levels of juiciness and fatness. Therefore, these results confirm the correlations between the marbling attribute and the texture parameters previously described by Hernández-Ramos et al. [8], which, as mentioned above, were attributed to the higher intramuscular fat content of the 50% Iberian samples, which increases juiciness and decreases hardness and fibrousness [42]. In addition, previous studies found a close relationship between the fat content and the sensory measurements of fat [35,43]. Furthermore, proteolytic changes in myofibrillar and sarcoplasmic proteins are crucial for the development of texture and sensory characteristics [44]. This process is affected by many factors, such as breeding conditions, the characteristics of the raw product, and the maturation process [45], but also by pig genetics [46] and lipid oxidation [47], as observed in this study.

### 3.2. Spectral Characteristics of the Samples Analyzed

**Figure 1:**
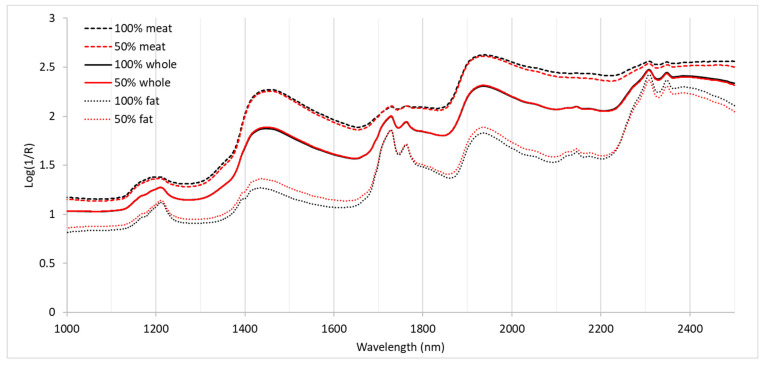
Mean spectra for 100% Iberian and 50% Iberian hams recorded using the BüchiNIRFlex N-500 device in the three areas studied (lean meat, fat, and whole slices).

Previous studies have shown that, although distinctive characteristics and differences can be observed between the spectra measured by benchtop and miniaturized spectrometers [48], the shapes of the absorption lines obtained with different equipment show similarities [23,49]. Therefore, Figure 1 shows the average spectra of 100% Iberian and 50% Iberian hams recorded for the lean meat, the fatty part of the slices, and the spectra of the whole slices, calculated as the average value of the spectra obtained for the fat and lean meat [27] by the NIRFlex N-500 equipment, which has the widest spectral range.

Differences owing to the breed of pig are more easily observed in the fat spectra, as in lean meat, they are less clear, and in whole slices, they are barely perceptible. In the first case, the 50% Iberian samples showed higher absorbance in the ranges between 1000 and 1600 nm and between 1800 and 2000 nm, which could be related to the higher fat content of the 50% Iberian samples, while in the 2100–2500 nm range, the 100% Iberian samples showed slightly higher absorbance. However, the 100% Iberian samples showed higher absorbance for all wavelengths when the spectra were recorded in lean meat.

The maximum absorbance peaks observed for the three recording sites at 1200 nm correspond to the second overtone of the C-H stretching vibration [50], and the peaks observed at 1730 and 1762 correspond to the first C-H stretching overtone. The lack of clear differences observed for these peaks corresponding to fatty acid composition could be due to the high number of shared absorbing molecular groups present in these compounds [51,52]. The combination tones of the C-H stretching and bending modes appear at 2310 and 2350 nm [53]. The NIR region between 2000 and 2300 is where the distinction between saturated and unsaturated compounds can most clearly be observed, as the specificity of the combined bands of unsaturated acids is manifested in this region [54]. The maximum peaks observed at 1450 and 1950 nm would be related to the -OH groups corresponding to the third, second, and first overtones of the stretching mode [55]. However, within this range, it is also possible to find N-H absorption bands around 1500, 1950, and 2450 nm, but most of them are rather difficult to discern, owing to their proximity to the absorption bands of fat and water, as the bands are very broad [53].

As regards the correlation between spectral characteristics and sensory parameters, the work of Hernández-Ramos et al. [8] showed a correlation between absorbance in the vicinity of 1500 nm and texture parameters such as hardness (1502, 1518, and 1530), fibrousness (1518 and 1528), and heterogeneity (1510, 1534), but also for 1720 and fibrousness, with all these parameters being significantly higher in the 100% Iberian samples. These wavelengths are related to the SH-SH bond (1736 nm) or to the absorption of amines, amides, urea, or proteins, and the 100% Iberian samples showed slightly higher absorbance when the spectra were taken from lean meat. On the other hand, the 50% Iberian samples were characterized by a greater sensation of fat, which showed a correlation with 1162 and 1720 nm, which, in turn, are correlated with C=O and CO-Oil bonds, but also with 1488 and 1520 nm, which correspond to amides, amines, and urea. The 50% Iberian samples showed significantly higher fatness values and slightly higher absorbance at 1500 and 1700 nm in the spectra of fat and the whole slices.

### 3.3. Prediction of Sensory Parameters

As previously reported, sensory characteristics are complex traits that require non-linear statistical approaches [56], such as artificial neural networks, which have proven to be a very suitable tool for meat products [8,19], as they provide better R^2^ than multiple regression tools [18]. Taking into account the results of previous studies on Iberian ham, a feedforward MLP ANN device with the Levenberg–Marquardt backpropagation training algorithm was used to predict the sensory parameters. In addition, 100 training times and a maximum number of neurons in the hidden layer of 15 were tested. Previous studies revealed that the greater the number of training sessions, the lower the number of neurons in the hidden layer required to obtain the highest R^2^. Therefore, for such a high number of training sessions, it would not be necessary to test a larger number of neurons. The best ANN architecture was selected based on the highest R^2^ values and the lowest RMSE values (training, validation, test, and total) (Table 2, Table 3 and Table 4). R^2^_Train_ indicates the way in which the network is able to predict results using the data with which it has been trained, R^2^_Valid_ gives information about the network’s overfitting, R^2^_Test_ measures the performance of the network and its ability to predict unknown data, and R^2^_Total_ measures the result of analyzing the data from all samples obtained through the network. Therefore, in this study, the R^2^_Test_ was prioritized, as it is more useful for evaluating the performance of the network once it is operational, and subsequently the R^2^_Total_, R^2^_Train_, and R^2^_Valid_.

The joint analysis of the results obtained for all parameters, devices, and recording sites revealed that R^2^_Train_ showed very high values, almost always above 0.8 and very often above 0.9. This is because during the training process, the errors between the data predicted and observed decrease until they reach a stable minimum. The network is then able to predict the training data with great accuracy. To calculate R^2^_Valid_, the network is tested with data that do not belong to the training set. After several passes through the training set data, the network is tested with data from the validation set, which was not used during training. As long as the validation errors continue to decrease, the network is learning. If, on the contrary, and as generally observed in this study, the errors increase during the validation process, this means that the network is overfitting. Therefore, the low values obtained in general for R^2^_Valid_ showed that the networks were learning but had problems in generalizing their performance to other data, i.e., they were overfitting. As for the R^2^_Test_, this parameter tended to have high values, as the ANN architecture was selected in accordance with it. Since R^2^_Total_ is the result of all the data passing through the network obtained, in general, satisfactory values were observed. It should also be noted that for some ANN architectures, apparently contradictory results were obtained, in which, although very good values were obtained during training, the validation values were very low, with the remainder of the values within a very high range. This was due to the small number of samples (60), which were divided into three data sets at random so that the validation and test sets included nine samples (15% of the samples). The results were therefore strongly affected by the type of samples included in each set for that ANN.

#### 3.3.1. Prediction of Sensory Parameters Using Spectra Recorded in Lean Meat

Six spectra were recorded at different points of the lean meat part of the slices by the BüchiNIRFlex N-500, MicroNIR 1700 ES, Enterprise Sensor, and microPHAZIRand SCiO devices, and the average spectrum for each sample was calculated and used for ANN analysis. The correlation coefficients (R^2^) and root mean square error (RMSE) for each device and sensory parameter are shown in Table 2. The results of the appearance, flavor, and texture parameters are presented separately.

The following levels of interpretations were used as a reference: R^2^ > 0.9 was considered excellent, R^2^ > 0.8 as good, R^2^ > 0.7 as acceptable, R^2^ > 0.6 as moderate, and R^2^ > 0.5 as fair. In addition to this, according to Williams [57], a calibration with an RSQ of <0.5 is considered unsuccessful, while an RSQ of between 0.50 and 0.65 indicates the possibility of distinguishing between high and low values. Therefore, for this study, a minimum RSQ value of >0.5 was chosen, as it points to the possibility of a successful calibration and can also be considered as an initial reference for comparing the feasibility of the prediction of the different devices used. On the other hand, an RSQ of 0.5–0.6 can be considered prospective. In this sense, high values for some attributes, such as the juiciness, softness, cured flavor, flavor intensity, and sweetness, are valued as positive, while high fibrousness, mold odor, and saltiness are valued as negative by consumers [4,58]. Therefore, high or low values for these parameters can be correlated with the acceptability of the cured ham, bearing in mind that this correlation is not always linear.

As regards appearance parameters (Table 2 (a)), all devices showed an R^2^_Total_ of >0.5 for all parameters, except microPHAZIR for color intensity. However, the number of parameters with an R^2^_total_ of >0.7 was lower and depended on the device. It was thus possible to predict four out of six parameters with NIRFlex N-500 (veined, fat color, color homogeneity, and color intensity), with the former two showing an R^2^_total_ of >0.8. For MicroNIR, veined, color homogeneity, and color intensity showed an R^2^_Total_ of >0.7, with the same color homogeneity for microPHAZIR, and only veined for Enterprise. Therefore, veined and color homogeneity were the parameters that reached the highest R^2^_Total_ values for this recording site, while the exudate showed the lowest values. It should be noted that the color homogeneity and exudate tended to have a higher number of neurons in the hidden layer, while the Enterprise device tended to show fewer neurons.

A total of 14 flavor parameters were analyzed (Table 2 (b)), and the results showed that 10 parameters for SCiO and 13 parameters for NIRFlex N-500 (all except aftertaste) were predicted with an R^2^_Total_ of >0.5. As with the appearance, the number of flavor parameters that showed an R^2^_Total_ of >0.7 was much lower, ranging from six for NIRFlex N-500 and microPHAZIR, including rancidity or sweetness with an R^2^_Total_ of >0.8, to four for MicroNIR (sweetness, R^2^_Total_ of >0.8), Enterprise (aftertaste, R^2^_total_ of >0.8), and SCiO (pig aroma, R^2^_total_ of >0.8). Flavor intensity was the best-predicted parameter, as all devices showed an R^2^_Total_ of >0.7, followed by pig aroma, while rancidity showed the worst results, as only NIRFlex N-500 and microPHAZIR showed an R^2^_Total_ of >0.5. For odor intensity, atypical aroma, and cured flavor, none of the devices showed an R^2^_Total_ of >0.7, although almost all of these showed an R^2^_Total_ of >0.5. In this case, in general, the cured aroma and atypical aroma showed a higher number of neurons in the hidden layer, while Enterprise and MicroNIIR tended to have a lower number of neurons.

Finally, eight texture parameters were also calibrated (Table 2 (c)). All devices were able to predict seven out of eight texture parameters with an R^2^_Total_ of >0.5, except for microPHAZIR, which only showed this R^2^_Total_ value for four parameters. For this group of sensory attributes, NIRFlex N-500, MicroNIR, and SCiO showed an R^2^_Total_ of >0.7 for four parameters and an R^2^_total_ of >0.8 for three parameters in the case of NIRFlex N-500 or two parameters for MicroNIR and SCiO (hardness, juiciness, and fibrousness in general). Hardness, juiciness, fatness, and fibrousness were predicted by all the devices with an R^2^_Total_ of >0.5, with fibrousness and juiciness having the highest R^2^_Total_ coefficients, while chewiness and homogeneity were predicted by all the devices except microPHAZIR. On the other hand, the parameter with the poorest prediction was chewing residue, as only NIRFlex N-500 showed an R^2^_Total_ of >0.5. This attribute also showed the highest number of neurons in the hidden layer. Overall, the number of parameters with an R^2^_Total_ of >0.5 was higher for appearance attributes, although the texture parameters showed slightly higher R^2^_Total_ values for this recording site.

Similar results were observed when RPD was considered (Table S3). NIRFlex N500, therefore, showed the highest number of parameters with high RPD values, with the Enterprise device having, in general, the lowest values for this parameter, followed by the SCiO device.

#### 3.3.2. Prediction of Sensory Parameters Using Spectra Recorded on Fat

As described above, for the spectral recording of fat, four spectra were taken from the fatty areas within the slices, and the average value of each sample was calculated. The results of calibration with ANN using sensory scores determined by the evaluators as reference analysis are shown in Table 3. For this recording site, it can be observed that all the parameters (except exudate for NIRFlex N-500, which showed an R^2^_total_ of 0.565) were predicted with an R^2^_Total_ of >0.6. MicroPHAZIR showed an R^2^_Total_ of >0.8 for three parameters (veined, the fat color, and white dots), while NIRFlex N-500 obtained these results for only two (color intensity and white dots), and Enterprise for only one, exudate (Table 3 (a)). In this case, the best-predicted parameters were, in general, white dots with an R^2^_total_ of >0.7 for most devices, followed by the fat color, while the exudate showed a higher R^2^_total_ than those observed when the calibration was made on lean meat, as the R^2^_totals_ were higher than 0.6 for most of the devices.

As for the odor and flavor parameters (Table 3 (b)), the NIRFlex N-500, microPHAZIR, and Enterprise devices were able to predict 13 out of 14 parameters with an R^2^_total_ of >0.5, while MicroNIR showed this R^2^_total_ value for 12 parameters, and SCiO only for 8 parameters. It is worth mentioning the best performance of NIRFlex N-500 for this set of parameters, as it showed an R^2^_total_ of >0.7 for six parameters (the pig aroma, rancid aroma, atypical aroma, fat flavor intensity, sweetness, and aftertaste) and an R^2^_total_ of >0.8 for two. The remainder of the devices showed an R^2^_total_ of >0.7 between four parameters and one parameter, with the latter being the SCiO equipment, which showed the poorest results among the devices used. The parameters that were predicted by a higher number of devices with the highest R^2^_total_ values were the atypical aroma, followed by the pig aroma, aftertaste, and fat flavor intensity. On the other hand, the lowest R^2^_totals_ were obtained for rancid and cured aromas.

As far as texture parameters are concerned (Table 3 (c)), the most relevant result was that all devices predicted hardness with an R^2^_Total_ of >0.8, reaching values of >0.9 for NIRFlex N-500 and microPHAZIR. These high R^2^_Total_ values were the result of very high values also for R^2^_Train_, R^2^_Valid_, and R^2^_Test_, which reveals the feasibility of predicting this sensory parameter by NIRS spectroscopy. Juiciness, fibrousness, and fatness also attained high R^2^_Total_ values of between 0.7 and 0.8 for all the devices except for SCiO (fatness). In contrast, gumminess showed very low R^2^_total_ values, with an R^2^_Total_ of >0.5 only observed for NIRFlex N-500 and microPHAZIR.

The best prediction performance was shown by NIRFlex N-500, as all the texture parameters were predicted with an R^2^_total_ of >0.6, with six of them with an R^2^_total_ of >0.7 and two of them with an R^2^_Total_ of >0.8, closely followed by microPHAZIR, which also predicted all the parameters with an R^2^_Total_ of >0.6. On the other hand, SCiO was only able to predict four out of eight values with an R^2^_total_ of >0.6 (six parameters with an R^2^_total_ of >0.5), and only hardness showed an R^2^_Total_ of >0.8.

These results demonstrated the poorer performance of the SCiO device when the spectra were recorded in the fatty areas of the ham slices, and that, in general, texture parameters were better predicted than appearance and flavor parameters for this recording site. These results were also observed when RPD was considered to assess the model performance (Table S3). Furthermore, no clear trend was observed in terms of the number of neurons in the hidden layer; neither of the parameters with a lower R^2^_Total_ showed a higher number, nor was there any device that tended to show a lower (or higher) number of neurons.

#### 3.3.3. Prediction of Sensory Parameters Using the Whole Slice Spectra

Finally, since fat and lean meat are consumed together, the spectrum of the whole slice was calculated for each sample as the average of the ten spectra recorded for the slices, except in the case of the Foss 5000. The calibration results, correlation coefficients (R^2^), and the root mean square error (RMSE) are shown in Table 4.

As was observed for the lean meat and fat recording sites, the number of calibrated parameters depended largely on the device. NIRFlex N-500, MicroNIR, and Enterprise therefore showed an R^2^_Total_ of >0.5 for all the appearance parameters (Table 4 (a)), while Foss 5000 and microPHAZIR showed this value for five out of six parameters, and SCiO showed this value only for three parameters. The number of parameters showing an R^2^_Total_ of >0.7 ranged from five (microPHAZIR) to one (SCiO and Foss 5000). However, it should be noted that NIRFlex N-500 showed an R^2^_Total_ of >0.8 for three parameters, which was the highest value among the devices used. The best performance of this equipment was corroborated by the higher RPD values observed for this set of sensory characteristics. The parameters that showed the highest R^2^_Totals_ for all the devices tested were veined, followed by color intensity, which was predicted by all of them, and color homogeneity. On the other hand, the exudate showed the lowest R^2^_total_ values and the highest number of neurons in the hidden layer.

In terms of flavor attributes (Table 4 (b)), NIRFlex N-500 again showed the highest number of predicted parameters (13), followed by microPHAZIR and MicroNIR (10), while SCiO showed the lowest performance. In fact, NIRFlex N-500 showed an R^2^_total_ of >0.7 for eight parameters, while the other devices showed a maximum of four parameters with this value. The best-predicted parameters were the saltiness, followed by the aftertaste and odor intensity, which were predicted by all the devices, while the poorest results were observed for rancidity, as only NIRFlex N-500 showed an R^2^_total_ of >0.5.

The results of texture parameter prediction (Table 4 (c)) revealed that NIRFlex N-500 showed an R^2^_total_ of >0.6 for the eight parameters analyzed and an R^2^_total_ of >0.8 for five of them. On the other hand, MicroNIR and SCiO had an R^2^_total_ of >0.5 for all the parameters. However, MicroNIR showed an R^2^_total_ of >0.7 for six parameters, while SCiO had this value for only four parameters. For this set of parameters, the Enterprise equipment showed the lowest prediction capacity, as only five parameters had an R^2^_total_ of >0.5. Fibrousness was the best-predicted parameter, followed by juiciness and heterogeneity, while gumminess and chewing residue displayed the lowest R^2^_total_ values. In this case, no clear trend regarding the number of neurons in the hidden layer was observed. Therefore, according to the RPD value, for this recording site, the best performance was observed for NIRFlex 500, while Foss 5000 did not show high RPD values. The high RPD values of the MicroPHAZIR device, followed by the MicroNIR device, are noteworthy.

The R^2^ values were generally lower than those previously reported for ham when whole slices were tested [8,21], and they were also lower than the R^2^ obtained for other meat products, such as dry-cured beef or loin [19,20], owing to the lower number and variability of the samples analyzed in this study and the lack of previous homogenization. For all recording sites and devices, in general, the highest R^2^ values were observed for texture parameters and the lowest R^2^ values were found for flavor parameters, which is in agreement with the results reported by Revilla et al. [19] and Curto et al. [59], while portable devices showed similar R^2^ values for both appearance and texture parameters, as reported by Vasconcelos et al. [20].

Table 5 summarizes the total number of parameters predicted by each device used, depending on the fixed R^2^_Total_ (>0.5, >0.6, >0.7, or >0.8), grouped by appearance, flavor, or texture attributes.

It can be observed that the best recording site depended strongly on the device. The NIRFlex N-500 benchtop device gave the best performance among all the devices used, which coincides with the previous results [27,49,60,61] for all recordings, with particularly high values for the entire slice registration methodology, with 27 of the 28 parameters predicted with an R^2^_Total_ of >0.5 for all recording sites and 18 parameters showing an R^2^_total_ of >0.7. These results mean that this benchtop equipment could be considered as a reference. However, the FOSS 5000 benchtop device did not perform as well as the previous one, with 20 parameters showing an R^2^_Total_ of >0.5 and only two parameters showing R^2^_Total_ values of >0.8, i.e., both microPHAZIR and MicroNIR achieved better results.

As for portable devices, when the spectra were recorded for whole slices, the MicroNIR performed very well, with 25 parameters showing an R^2^_total_ of >0.5 and 13 parameters reaching an R^2^_total_ of >0.7. When the spectra were recorded for fat, microPHAZIR and Enterprise showed the highest number of parameters predicted, with 27 and 26 parameters, respectively, predicted with an R^2^_total_ of >0.5 and 13 parameters predicted with an R^2^_total_ of >0.7 in both cases. On the other hand, MicroNIR and SCiO demonstrated better performance when spectra were recorded for lean meat with 25 and 23 parameters, with an R^2^_Total_ of >0.5 and 11 and 10 parameters with an R^2^_Total_ of >0.7, respectively. These results differ, in general, from those previously reported for fatty acids, which were better predicted for lean meat except in the case of SCIO, which showed better performance for fat [27].

The results, therefore, suggest that, in general, the parameters predicted with the highest R^2^_total_ depended on the recording area, except for MicroNIR, which showed very consistent results for all sites, which indicates that measurements taken in different areas provide complementary information, as previously reported by Hernández-Jiménez et al. [24]. Accordingly, NIRFlex N-500 was able to predict a total of 16 different sensory parameters with an R^2^_Total_ of >0.8 (veined, fat color, color intensity, color homogeneity, white dots, rancidity, typical aroma, cured flavor, cured aroma, saltiness, hardness, juiciness, fatness, fibrousness, chewiness, and heterogeneity). Among the portable devices, microPHAZIR showed a total of nine parameters (veined, color homogeneity, fat color, sweetness, aftertaste, cured aroma, hardness, juiciness, and fibrousness), Enterprise showed eight parameters (veined, exudate, aftertaste, cured flavor, saltiness, hardness, juiciness, and fibrousness), MicroNIR1700 showed a total of seven parameters (veined, color homogeneity, fat color, sweetness, sourness, hardness, and juiciness), and SCiO showed a total of four different attributes (pig aroma, atypical aroma, saltiness, and hardness) when using the information from all the recording possibilities. It is worth highlighting that some of these characteristics, such as hardness, fibrousness, juiciness, color homogeneity, flavor intensity, sweetness, and fat color, are decisive in consumer acceptance of Iberian ham [4,58].

## 4. Conclusions

These results allowed us to conclude that, on the one hand, when using a portable device, it is essential to optimize the recording area, but, on the other hand, the results obtained at different recording sites are complementary. Among the sensory parameters analyzed in this study, veins, color homogeneity, color intensity, hardness, juiciness, fatness, and fibrousness showed statistically significant differences owing to the breed, which makes them particularly relevant to the characterization of Iberian ham. These parameters were predicted by all devices and at all recording sites. The best results were obtained with the NIRFlex N-500, with between 26 and 27 out of the 28 parameters predicted. Among all the portable devices used, MicroNIR and microPHAZIR gave the best results, as they were able to predict the aforementioned parameters with high R^2^ values in addition to the parameters of flavor intensity, sweetness, and fat color, which are decisive for consumer acceptance of Iberian ham. These results point to the suitability of portable devices for predicting the sensory properties of Iberian ham. However, a larger set of samples with greater variability must be analyzed to improve the performance of the ANN, especially the R^2^ values of the validation set. This would allow a broader application of the methods developed.

## Figures and Tables

**Table 1:** Mean value and statistical descriptors of each sensory attribute for the two genetic types.

		100% Iberian	Iberian	SEM	Min	Max	CV	*p*-Value
Appearance	Veined	4.96	6.11	0.10	2.75	7.50	0.14	0.000
	Fat color	7.04	6.86	0.54	5.56	7.71	0.04	0.130
	Color homogeneity	5.59	5.98	0.64	4.67	6.88	0.06	0.002
	Color intensity	7.33	6.96	0.06	5.89	8.31	0.04	0.002
	Exudate	5.03	5.08	0.07	4.13	6.13	0.07	0.704
	White dots	2.87	3.40	0.10	2.00	5.25	0.17	0.005
Flavor	Odor intensity	6.67	6.75	0.04	6.00	7.33	0.03	0.308
	Cured aroma	6.75	6.78	0.03	5.94	7.28	0.03	0.740
	Pig aroma	1.29	1.27	0.15	1.00	1.63	0.06	0.490
	Rancid aroma	1.52	1.62	0.03	1.22	2.17	0.08	0.050
	Atypical aroma	1.37	1.40	0.02	1.13	2.00	0.08	0.401
	Flavor intensity	6.77	6.83	0.04	6.17	7.38	0.03	0.417
	Fat flavor intensity	4.83	5.08	0.07	3.88	6.44	0.07	0.058
	Cured flavor	6.67	6.66	0.04	5.88	7.25	0.03	0.903
	Saltiness	4.29	4.23	0.05	3.50	5.25	0.06	0.587
	Sweetness	2.48	2.48	0.03	1.88	3.00	0.07	0.909
	Sourness	1.40	1.42	0.03	1.11	2.13	0.09	0.819
	Rancidity	1.61	1.66	0.03	1.11	2.25	0.10	0.516
	Aftertaste	5.95	6.06	0.05	5.25	6.75	0.04	0.274
	Atypical flavor	1.46	1.45	0.04	1.00	2.13	0.13	0.882
Texture	Hardness	4.54	4.03	0.07	3.28	6.25	0.09	0.001
	Juiciness	5.72	6.03	0.06	4.75	6.71	0.05	0.009
	Fatness	4.61	4.92	0.06	3.57	6.14	0.07	0.019
	Fibrousness	3.50	3.17	0.07	2.43	5.00	0.10	0.020
	Chewiness	3.24	3.10	0.05	2.43	4.38	0.08	0.204
	Gumminess	2.37	2.44	0.04	1.75	3.13	0.09	0.430
	Heterogeneity	3.80	3.41	0.07	2.43	4.75	0.10	0.005
	Chewing Residue	2.73	2.72	0.04	2.22	3.50	0.08	0.832

SEM: standard error mean. Min: minimum value. Max: maximum value. CV: coefficient of variation.

**Table 2:** (**a**). Correlation coefficients (R^2^) and root mean square error (RMSE) values for the best ANN obtained from the NIR spectra of lean meat: Appearance parameters; (**b**). Correlation coefficients (R^2^) and root mean square error (RMSE) values for the best ANN obtained from the NIR spectra of lean meat: Flavor attributes; (**c**). Correlation coefficients (R^2^) and root mean square error (RMSE) values for the best ANN obtained from the NIR spectra of lean meat: Texture attributes.

(**a**)																																										
				**Veined**											**Fat Color**													**Color Homogeneity**														
				N		R^2^_Train_		R^2^_Valid_		R^2^_Test_			R^2^_Total_		N				R^2^_Train_		R^2^_Valid_			R^2^_Test_		R^2^_Total_		N		R^2^_Train_			R^2^_Valid_			R^2^_Test_			R^2^_Total_			
						RMSE		RMSE		RMSE			RMSE						RMSE		RMSE			RMSE		RMSE				RMSE			RMSE			RMSE			RMSE			
NIRFlex N-500				5		0.956		0.613		0.730			0.819		8				0.997		0.764			0.662		0.855		13		0.999			0.553			0.505			0.756			
						0.220		0.744		0.827			0.469						0.025		0.174			0.385		0.165				0.014			0.418			0.502			0.253			
microPHAZIR				9		0.926		0.520		0.642			0.802		8				0.978		0.914			0.516		0.666		11		1.000			0.535			0.701			0.801			
						0.346		0.796		0.686			0.500						0.058		0.518			0.352		0.247				0.008			0.502			0.273			0.221			
MicroNIR 1700				8		0.886		0.887		0.720			0.829		13				0.977		0.808			0.558		0.657		15		0.989			0.724			0.587			0.891			
						0.409		0.371		0.643			0.447						0.062		0.376			0.820		0.353				0.052			0.279			0.297			0.164			
Enterprise				1		0.980		0.500		0.393			0.819		6				0.814		0.338			0.650		0.647		15		0.862			0.725			0.523			0.634			
						0.235		0.614		0.895			0.464						0.190		0.351			0.381		0.256				0.180			0.436			0.630			0.333			
SCiO				11		0.968		0.730		0.759			0.783		6				0.634		0.600			0.771		0.548		12		0.702			0.427			0.499			0.577			
						0.196		0.645		1.324			0.594						0.253		0.404			0.292		0.286				0.286			0.400			0.361			0.318			
				**Color intensity**											**Exudate**													**White dots**														
				N		R^2^_Train_		R^2^_Valid_		R^2^_Test_			R^2^_Total_		N				R^2^_Train_		R^2^_Valid_			R^2^_Test_		R^2^_Total_		N		R^2^_Train_			R^2^_Valid_			R^2^_Test_			R^2^_Total_			
						RMSE		RMSE		RMSE			RMSE						RMSE		RMSE			RMSE		RMSE				RMSE			RMSE			RMSE			RMSE			
NIRFlex N-500				6		0.868		0.335		0.371			0.775		4				0.593		0.561			0.561		0.545		10		0.790			0.310			0.432			0.603			
						0.201		0.261		0.279			0.224						0.320		0.295			0.456		0.341				0.335			0.722			0.775			0.497			
microPHAZIR				6		0.908		0.533		0.459			0.612		10				0.674		0.436			0.517		0.458		14		0.678			0.139			0.585			0.558			
						0.169		0.264		0.613			0.295						0.332		0.385			0.569		0.385				0.441			0.779			0.568			0.525			
MicroNIR 1700				9		0.823		0.511		0.857			0.707		12				0.811		0.222			0.339		0.525		13		0.999			0.532			0.653			0.699			
						0.197		0.510		0.198			0.268						0.226		0.498			0.618		0.361				0.025			0.550			1.092			0.474			
Enterprise				3		0.791		0.350		0.456			0.618		15				0.844		0.213			0.400		0.571		1		0.982			0.051			0.433			0.690			
						0.232		0.405		0.405			0.295						0.199		0.640			0.397		0.336				0.295			0.927			0.602			0.494			
SCiO				5		0.622		0.370		0.476			0.509		14				0.780		0.488			0.366		0.545		14		0.964			0.916			0.551			0.758			
						0.284		0.332		0.534			0.340						0.238		0.494			0.666		0.378				0.126			0.542			0.747			0.373			
(**b**)																																										
			**Odor Intensity**														**Cured aroma**											**Pig aroma**														
			N			R^2^_Train_		R^2^_Valid_		R^2^_Test_			R^2^_Total_			N			R^2^_Train_		R^2^_Valid_			R^2^_Test_		R^2^_Total_		N		R^2^_Train_			R^2^_Valid_				R^2^_Test_			R^2^_Total_		
						RMSE		RMSE		RMSE			RMSE						RMSE		RMSE			RMSE		RMSE				RMSE			RMSE				RMSE			RMSE		
NIRFlex N-500			10			0.849		0.002		0.688			0.559			12			0.867		0.616			0.484		0.655		7		0.933			0.488				0.550			0.655		
						0.113		0.364		0.204			0.187						0.090		0.199			0.340		0.170				0.036			0.130				0.093			0.069		
microPHAZIR			8			0.821		0.100		0.502			0.614			10			0.906		0.754			0.561		0.725		13		0.953			0.207				0.638			0.750		
						0.118		0.286		0.252			0.178						0.084		0.152			0.298		0.147				0.031			0.117				0.067			0.058		
MicroNIR 1700						0.974		0.018		0.531			0.532			4			0.995		0.502			0.306		0.572		8		0.801			0.413				0.479			0.700		
						0.045		0.348		0.436			0.219						0.028		0.253			0.444		0.199				0.056			0.076				0.089			0.065		
Enterprise			1			0.903		0.410		0.393			0.573			14			0.708		0.765			0.565		0.657		10		1.000			0.264				0.745			0.778		
						0.089		0.226		0.370			0.184						0.131		0.155			0.221		0.152				0.000			0.132				0.098			0.064		
SCiO			11			0.811		0.243		0.613			0.583			14			0.892		0.573			0.621		0.728		13		0.970			0.460				0.736			0.859		
						0.113		0.333		0.247			0.186						0.094		0.163			0.269		0.145				0.022			0.078				0.073			0.045		
			**Rancid aroma**														**Atypical aroma**											**Flavor intensity**														
			N			R^2^_Train_		R^2^_Valid_		R^2^_Test_			R^2^_Total_			N			R^2^_Train_		R^2^_Valid_			R^2^_Test_		R^2^_Total_		N		R^2^_Train_			R^2^_Valid_				R^2^_Test_			R^2^_Total_		
						RMSE		RMSE		RMSE			RMSE						RMSE		RMSE			RMSE		RMSE				RMSE			RMSE				RMSE			RMSE		
NIRFlex N-500			7			0.988		0.462		0.859			0.795			1			0.849		0.591			0.665		0.582		1		0.960			0.594				0.437			0.722		
						0.019		0.215		0.106			0.094						0.056		0.176			0.184		0.110				0.086			0.208				0.292			0.156		
microPHAZIR			15			0.917		0.492		0.337			0.550			14			0.998		0.097			0.403		0.610		12		1.000			0.105				0.411			0.727		
						0.060		0.175		0.256			0.130						0.009		0.121			0.262		0.112				0.000			0.318				0.242			0.155		
MicroNIR 1700			9			0.804		0.181		0.444			0.394			12			0.876		0.528			0.409		0.673		13		0.984			0.473				0.537			0.751		
						0.092		0.212		0.338			0.173						0.063		0.140			0.174		0.101				0.039			0.236				0.295			0.150		
Enterprise			5			0.781		0.507		0.411			0.598			14			0.899		0.544			0.395		0.554		7		0.983			0.691				0.625			0.730		
						0.099		0.185		0.152			0.124						0.060		0.100			0.248		0.115				0.035			0.235				0.315			0.155		
SCiO			5			0.512		0.475		0.471			0.472			11			0.543		0.225			0.454		0.473		12		0.816			0.425				0.400			0.715		
						0.150		0.104		0.145			0.143						0.127		0.127			0.110		0.125				0.135			0.217				0.186			0.158		
		**Fat flavor intensity**									**Cured flavor**											**Saltiness**									**Sweetness**											
		N	R^2^_Train_		R^2^_Valid_		R^2^_Test_		R^2^_Total_		N	R^2^_Train_		R^2^_Valid_				R^2^_Test_		R^2^_Total_		N	R^2^_Train_		R^2^_Valid_		R^2^_Test_		R^2^_Total_		N	R^2^_Train_			R^2^_Valid_			R^2^_Test_			R^2^_Total_	
			RMSE		RMSE		RMSE		RMSE			RMSE		RMSE				RMSE		RMSE			RMSE		RMSE		RMSE		RMSE			RMSE			RMSE			RMSE			RMSE	
NIRFlex N-500		14	1.000		0.176		0.469		0.651		9	0.687		0.162				0.803		0.627		9	0.995		0.487		0.626		0.741		8	0.825			0.276			0.783			0.676	
			0.000		0.803		0.598		0.388			0.174		0.178				0.254		0.189			0.027		0.401		0.340		0.205			0.105			0.278			0.148			0.150	
microPHAZIR		7	0.891		0.468		0.653		0.788		15	0.864		0.293				0.173		0.482		7	0.994		0.424		0.301		0.626		1	1.000			0.363			0.768			0.884	
			0.172		0.345		0.378		0.245			0.106		0.438				0.347		0.234			0.028		0.294		0.501		0.226			0.006			0.177			0.149			0.090	
MicroNIR 1700		10	0.664		0.615		0.617		0.614		15	0.999		0.445				0.426		0.644		9	0.773		0.722		0.778		0.738		6	0.929			0.814			0.735			0.833	
			0.316		0.355		0.331		0.324			0.008		0.344				0.497		0.234			0.159		0.213		0.238		0.182			0.068			0.102			0.229			0.112	
Enterprise		5	0.998		0.385		0.326		0.663		2	0.959		0.195				0.485		0.688		7	0.783		0.498		0.738		0.721		1	0.887			0.197			0.518			0.615	
			0.029		0.557		0.739		0.359			0.060		0.351				0.247		0.174			0.178		0.205		0.273		0.199			0.114			0.283			0.148			0.156	
SCiO		6	0.712		0.288		0.860		0.644		5	0.720		0.212				0.760		0.572		1	0.839		0.415		0.642		0.640		4	0.833			0.683			0.603			0.701	
			0.301		0.447		0.244		0.320			0.171		0.312				0.181		0.200			0.146		0.312		0.394		0.230			0.096			0.247			0.161			0.140	
		**Sourness**									**Rancidity**											**Aftertaste**									**Atypical flavor**											
		N	R^2^_Train_		R^2^_Valid_		R^2^_Test_		R^2^_Total_		N	R^2^_Train_		R^2^_Valid_				R^2^_Test_		R^2^_Total_		N	R^2^_Train_		R^2^_Valid_		R^2^_Test_		R^2^_Total_		N	R^2^_Train_			R^2^_Valid_			R^2^_Test_			R^2^_Total_	
			RMSE		RMSE		RMSE		RMSE			RMSE		RMSE				RMSE		RMSE			RMSE		RMSE		RMSE		RMSE			RMSE			RMSE			RMSE			RMSE	
NIRFlex N-500		5	0.944		0.276		0.486		0.701		9	0.975		0.376				0.691		0.828		9	0.754		0.299		0.725		0.359		2	0.962			0.451			0.686			0.727	
			0.053		0.221		0.157		0.114			0.043		0.195				0.160		0.104			0.185		0.365		0.551		0.299			0.052			0.293			0.216			0.148	
microPHAZIR		10	0.930		0.363		0.397		0.725		3	0.753		0.173				0.655		0.568		13	0.699		0.019		0.548		0.443		4	0.793			0.264			0.763			0.649	
			0.057		0.135		0.205		0.106			0.160		0.273				0.187		0.185			0.219		0.483		0.274		0.282			0.152			0.177			0.322			0.191	
MicroNIR 1700		5	0.911		0.418		0.514		0.586		12	0.811		0.206				0.582		0.174		11	0.731		0.297		0.816		0.502		7	0.880			0.211			0.682			0.582	
			0.061		0.159		0.291		0.138			0.105		0.438				0.415		0.250			0.193		0.291		0.467		0.267			0.113			0.245			0.350			0.191	
Enterprise		4	0.753		0.074		0.644		0.525		8	0.732		0.005				0.690		0.357		1	1.000		0.593		0.902		0.863		10	0.742			0.253			0.728			0.360	
			0.110		0.223		0.161		0.141			0.132		0.328				0.298		0.204			0.001		0.264		0.270		0.146			0.143			0.252			0.430			0.227	
SCiO		10	0.896		0.172		0.272		0.495		12	0.869		0.262				0.817		0.384		7	0.698		0.281		0.729		0.566		14	0.537			0.219			0.589			0.464	
			0.068		0.168		0.335		0.156			0.085		0.199				0.437		0.199			0.203		0.387		0.216		0.241			0.195			0.264			0.156			0.202	
(**c**)																																										
	**Hardness**										**Juiciness**											**Fatness**									**Fibrousness**											
	N		R^2^_Train_		R^2^_Valid_		R^2^_Test_		R^2^_Total_		N	R^2^_Train_		R^2^_Valid_				R^2^_Test_		R^2^_Total_		N	R^2^_Train_		R^2^_Valid_		R^2^_Test_		R^2^_Total_		N	R^2^_Train_		R^2^_Valid_				R^2^_Test_				R^2^_Total_
			RMSE		RMSE		RMSE		RMSE			RMSE		RMSE				RMSE		RMSE			RMSE		RMSE		RMSE		RMSE			RMSE		RMSE				RMSE				RMSE
NIRFlex N-500	7		0.836		0.841		0.878		0.843		11	0.980		0.688				0.806		0.885		13	0.934		0.223		0.777		0.578		15	1.000		0.532				0.840				0.862
			0.204		0.302		0.218		0.224			0.064		0.230				0.269		0.147			0.117		0.772		0.423		0.355			0.000		0.497				0.249				0.215
microPHAZIR	6		0.762		0.567		0.842		0.686		2	0.838		0.265				0.796		0.729		11	0.755		0.607		0.868		0.536		11	1.000		0.026				0.748				0.617
			0.257		0.603		0.264		0.334			0.166		0.363				0.308		0.231			0.271		0.304		0.584		0.341			0.000		0.747				0.459				0.339
MicroNIR 1700	2		1.000		0.699		0.889		0.897			0.918		0.791				0.834		0.851		3	0.687		0.472		0.804		0.658		8	0.804		0.560				0.746				0.714
			0.092		0.304		0.412		0.213			0.120		0.274				0.248		0.175			0.290		0.394		0.279		0.306			0.237		0.281				0.520				0.303
Enterprise	13		0.965		0.366		0.903		0.622		14	0.722		0.536				0.638		0.641		9	0.540		0.554		0.712		0.549		8	0.950		0.631				0.802				0.847
			0.109		0.619		0.826		0.410			0.270		0.371				0.344		0.299			0.408		0.574		0.619		0.473			0.126		0.260				0.409				0.215
SCiO	4		0.832		0.800		0.964		0.813		5	0.668		0.857				0.887		0.702		14	0.882		0.638		0.647		0.763		10	0.967		0.233				0.829				0.840
			0.195		0.395		0.347		0.261			0.267		0.174				0.118		0.238			0.217		0.438		0.306		0.275			0.106		0.364				0.373				0.220
	**Chewiness**										**Gumminess**											**Heterogeneity**									**Chewing residue**											
	N		R^2^_Train_		R^2^_Valid_		R^2^_Test_		R^2^_Total_		N	R^2^_Train_		R^2^_Valid_				R^2^_Test_		R^2^_Total_		N	R^2^_Train_		R^2^_Valid_		R^2^_Test_		R^2^_Total_		N	R^2^_Train_		R^2^_Valid_				R^2^_Test_				R^2^_Total_
			RMSE		RMSE		RMSE		RMSE			RMSE		RMSE				RMSE		RMSE			RMSE		RMSE		RMSE		RMSE			RMSE		RMSE				RMSE				RMSE
NIRFlex N-500	13		1.000		0.243		0.779		0.646		4	0.789		0.637				0.694		0.283		7	0.913		0.639		0.856		0.762		9	0.522		0.323				0.613				0.504
			0.008		0.708		0.313		0.300			0.143		0.162				0.678		0.295			0.143		0.488		0.325		0.257			0.225		0.261				0.195				0.226
microPHAZIR	10		0.860		0.312		0.782		0.348		4	0.873		0.237				0.614		0.418		1	1.000		0.322		0.773		0.426		12	0.650		0.059				0.608				0.359
			0.161		0.560		0.500		0.321			0.121		0.350				0.462		0.246			0.000		0.546		0.965		0.429			0.195		0.274				0.403				0.250
MicroNIR 1700	2		0.794		0.817		0.754		0.784		4	0.826		0.561				0.733		0.670		5	0.576		0.617		0.649		0.570		14	0.510		0.290				0.639				0.438
			0.171		0.252		0.170		0.185			0.131		0.220				0.391		0.205			0.304		0.454		0.373		0.341			0.237		0.241				0.256				0.241
Enterprise	8		0.735		0.343		0.676		0.672		12	0.803		0.506				0.614		0.678		1	0.744		0.369		0.714		0.661		11	0.998		0.300				0.667				0.335
			0.194		0.256		0.308		0.225			0.127		0.284				0.240		0.179			0.273		0.397		0.354		0.307			0.013		0.340				0.730				0.312
SCiO	14		0.704		0.681		0.641		0.662		8	0.773		0.116				0.722		0.550		4	0.670		0.614		0.764		0.643		12	0.741		0.339				0.790				0.358
			0.192		0.365		0.271		0.238			0.146		0.287				0.369		0.219			0.277		0.383		0.395		0.315			0.179		0.190				0.514				0.260

N: number of neurons in the hidden layer.

**Table 3:** (**a**). Correlation coefficients (R^2^) and root mean square error (RMSE) values for the best ANN obtained from the NIR spectra of fat: Appearance parameters; (**b**). Correlation coefficients (R^2^) and root mean square error (RMSE) values for the best ANN obtained from the NIR spectra of fat: Flavor attributes; (**c**). Correlation coefficients and root mean square error (RMSE) values for the best ANN architecture obtained from the NIR spectra of fat: Texture attributes.

(**a**)																																																							
					**Veined**																	**Fat Color**																	**Color Homogeneity**																
					N			R^2^_Train_			R^2^_Valid_			R^2^_Test_				R^2^_Total_				N			R^2^_Train_			R^2^_Valid_					R^2^_Test_			R^2^_Total_			N			R^2^_Train_					R^2^_Valid_			R^2^_Test_			R^2^_Total_		
								RMSE			RMSE			RMSE				RMSE							RMSE			RMSE					RMSE			RMSE						RMSE					RMSE			RMSE			RMSE		
NIRFlex N-500					7			0.851			0.395			0.678				0.745				6			0.704			0.635					0.666			0.654			5			0.852					0.474			0.498			0.729		
								0.427			0.876			0.679				0.559							0.214			0.401					0.221			0.252						0.199					0.344			0.258			0.377		
microPHAZIR					13			0.999			0.566			0.740				0.831				3			1.000			0.705					0.641			0.843			3			1.000					0.524			0.678			0.758		
								0.037			0.932			0.769				0.469							0.002			0.357					0.264			0.172						0.009					0.320			0.277			0.639		
MicroNIR 1700					5			0.738			0.314			0.800				0.660				11			0.854			0.655					0.540			0.716			3			0.851					0.694			0.466			0.706		
								0.508			0.908			0.806				0.634							0.169			0.204					0.401			0.225						0.194					0.430			0.282			0.412		
Enterprise					5			0.825			0.489			0.743				0.738				5			0.922			0.700					0.609			0.795			14			0.967					0.475			0.439			0.691		
								0.451			0.862			0.643				0.562							0.115			0.258					0.369			0.199						0.097					0.541			0.327			0.614		
SCiO					14			0.850			0.101			0.582				0.639				12			0.832			0.282					0.413			0.553			15			0.833					0.288			0.710			0.673		
								0.443			1.095			0.944				0.671							0.176			0.307					0.534			0.280						0.203					0.430			0.288			0.417		
					**Color intensity**																	**Exudate**																	**White dots**																
					N			R^2^_Train_			R^2^_Valid_			R^2^_Test_				R^2^_Total_				N			R^2^_Train_			R^2^_Valid_					R^2^_Test_			R^2^_Total_			N			R^2^_Train_					R^2^_Valid_			R^2^_Test_			R^2^_Total_		
								RMSE			RMSE			RMSE				RMSE							RMSE			RMSE					RMSE			RMSE						RMSE					RMSE			RMSE			RMSE		
NIRFlex N-500					9			0.992			0.355			0.610				0.815				11			0.970			0.235					0.493			0.565			13			0.917					0.778			0.548			0.822		
								0.045			0.382			0.421				0.223							0.094			0.685					0.579			0.356						0.239					0.333			0.559			0.322		
microPHAZIR					7			0.919			0.339			0.482				0.662				8			0.849			0.473					0.351			0.646			15			1.000					0.626			0.458			0.848		
								0.137			0.522			0.419				0.284							0.215			0.454					0.443			0.304						0.000					0.540			0.550			0.299		
MicroNIR 1700					11			0.588			0.675			0.624				0.601				10			0.843			0.292					0.652			0.652			1			0.883					0.628			0.781			0.703		
								0.315			0.280			0.247				0.301							0.218			0.458					0.514			0.323						0.372					0.522			1.015			0.540		
Enterprise					2			0.750			0.666			0.578				0.694				13			0.990			0.838					0.305			0.826			6			0.983					0.634			0.715			0.707		
								0.235			0.355			0.292				0.265							0.047			0.263					0.481			0.216						0.104					0.612			1.136			0.508		
SCiO					8			0.907			0.617			0.202				0.558				10			0.944			0.242					0.723			0.693			13			0.808					0.651			0.419			0.684		
								0.143			0.397			0.667				0.323							0.117			0.609					0.582			0.341						0.328					0.396			0.750			0.428		
(**b**)																																																							
					**Odor Intensity**																**Cured aroma**																		**Pig aroma**																
					N			R^2^_Train_			R^2^_Valid_			R^2^_Test_				R^2^_Total_			N				R^2^_Train_			R^2^_Valid_					R^2^_Test_			R^2^_Total_			N			R^2^_Train_					R^2^_Valid_			R^2^_Test_			R^2^_Total_		
								RMSE			RMSE			RMSE				RMSE							RMSE			RMSE					RMSE			RMSE						RMSE					RMSE			RMSE			RMSE		
NIRFlex N-500					6			0.857			0.366			0.729				0.596			4				0.781			0.723					0.472			0.676			6			0.869					0.140			0.811			0.734		
								0.112			0.361			0.238				0.192							0.106			0.184					0.246			0.148						0.049					0.100			0.121			0.073		
microPHAZIR					10			0.878			0.167			0.568				0.743			8				0.936			0.683					0.113			0.599			1			0.983					0.195			0.376			0.505		
								0.113			0.213			0.174				0.142							0.064			0.264					0.296			0.163						0.022					0.111			0.191			0.088		
MicroNIR 1700					11			0.800			0.403			0.449				0.609			11				0.649			0.219					0.271			0.460			11			0.877					0.537			0.547			0.730		
								0.134			0.158			0.346				0.185							0.173			0.221					0.225			0.189						0.037					0.090			0.118			0.066		
Enterprise					13			0.734			0.473			0.372				0.544			11				0.949			0.344					0.216			0.470			10			0.967					0.628			0.430			0.793		
								0.176			0.141			0.276				0.190							0.060			0.346					0.369			0.202						0.024					0.087			0.095			0.054		
SCiO					5			0.578			0.357			0.454				0.506			14				0.480			0.389					0.171			0.302			14			0.720					0.310			0.335			0.617		
								0.195			0.232			0.207				0.203							0.201			0.267					0.233			0.217						0.066					0.087			0.088			0.073		
					**Rancid aroma**																**Atypical aroma**																		**Flavor intensity**																
					N			R^2^_Train_			R^2^_Valid_			R^2^_Test_				R^2^_Total_			N				R^2^_Train_			R^2^_Valid_					R^2^_Test_			R^2^_Total_			N			R^2^_Train_					R^2^_Valid_			R^2^_Test_			R^2^_Total_		
								RMSE			RMSE			RMSE				RMSE							RMSE			RMSE					RMSE			RMSE						RMSE					RMSE			RMSE			RMSE		
NIRFlex N-500					15			0.990			0.072			0.708				0.331			1				1.000			0.445					0.652			0.863			15			0.974					0.588			0.344			0.602		
								0.021			0.240			0.420				0.188							0.000			0.135					0.099			0.065						0.046					0.232			0.515			0.222		
microPHAZIR					4			0.632			0.420			0.763				0.156			9				0.869			0.397					0.486			0.654			3			0.847					0.650			0.635			0.774		
								0.113			0.214			0.330				0.179							0.057			0.176					0.148			0.101						0.112					0.203			0.187			0.142		
MicroNIR 1700					11			0.627			0.591			0.403				0.568			12				0.638			0.603					0.682			0.609			5			0.700					0.302			0.349			0.442		
								0.134			0.097			0.139				0.130							0.114			0.084					0.088			0.106						0.163					0.255			0.472			0.249		
Enterprise					8			0.919			0.028			0.499				0.612			13				0.925			0.589					0.651			0.764			14			0.803					0.309			0.518			0.632		
								0.050			0.225			0.186				0.121							0.052			0.174					0.140			0.097						0.132					0.313			0.200			0.181		
SCiO					8			0.662			0.803			0.548				0.575			15				0.940			0.687					0.320			0.811			14			0.736					0.652			0.377			0.402		
								0.122			0.089			0.182				0.129							0.044			0.088					0.144			0.075						0.160					0.190			0.470			0.237		
	**Fat flavor intensity**														**Cured flavor**														**Saltiness**														**Sweetness**												
	N		R^2^_Train_			R^2^_Valid_			R^2^_Test_			R^2^_Total_			N		R^2^_Train_		R^2^_Valid_				R^2^_Test_			R^2^_Total_			N		R^2^_Train_			R^2^_Valid_			R^2^_Test_			R^2^_Total_			N		R^2^_Train_			R^2^_Valid_			R^2^_Test_			R^2^_Total_	
			RMSE			RMSE			RMSE			RMSE					RMSE		RMSE				RMSE			RMSE					RMSE			RMSE			RMSE			RMSE					RMSE			RMSE			RMSE			RMSE	
NIRFlex N-500	7		0.952			0.349			0.626			0.743			6		0.951		0.715				0.647			0.826			8		0.779			0.550			0.581			0.680			12		0.950			0.315			0.719			0.761	
			0.110			0.468			0.442			0.266					0.064		0.190				0.223			0.126					0.172			0.262			0.248			0.200					0.059			0.251			0.188			0.131	
microPHAZIR	9		0.702			0.047			0.652			0.608			12		0.794		0.546				0.797			0.692			15		0.969			0.813			0.273			0.600			9		0.968			0.228			0.508			0.597	
			0.338			0.449			0.376			0.363					0.126		0.270				0.225			0.172					0.060			0.294			0.495			0.228					0.048			0.401			0.173			0.174	
MicroNIR 1700	10		0.980			0.649			0.840			0.774			15		0.729		0.120				0.780			0.637			14		0.897			0.445			0.497			0.610			15		0.947			0.718			0.826			0.858	
			0.072			0.440			0.595			0.293					0.165		0.213				0.226			0.183					0.107			0.429			0.309			0.224					0.056			0.170			0.148			0.099	
Enterprise	14		0.994			0.393			0.369			0.679			7		0.759		0.383				0.448			0.654			12		0.729			0.244			0.625			0.615			8		0.699			0.499			0.884			0.660	
			0.041			0.463			0.657			0.313					0.147		0.228				0.257			0.181					0.199			0.334			0.144			0.218					0.148			0.200			0.188			0.164	
SCiO	11		0.717			0.171			0.453			0.513			9		0.782		0.460				0.366			0.502			12		0.556			0.153			0.639			0.459			5		0.578			0.541			0.277			0.468	
			0.280			0.442			0.736			0.407					0.129		0.244				0.396			0.210					0.236			0.336			0.265			0.258					0.176			0.118			0.268			0.186	
	**Sourness**														**Rancidity**														**Aftertaste**														**Atypical flavor**												
	N		R^2^_Train_			R^2^_Valid_			R^2^_Test_			R^2^_Total_			N		R^2^_Train_		R^2^_Valid_				R^2^_Test_			R^2^_Total_			N		R^2^_Train_			R^2^_Valid_			R^2^_Test_			R^2^_Total_			N		R^2^_Train_			R^2^_Valid_			R^2^_Test_			R^2^_Total_	
			RMSE			RMSE			RMSE			RMSE					RMSE		RMSE				RMSE			RMSE					RMSE			RMSE			RMSE			RMSE					RMSE			RMSE			RMSE			RMSE	
NIRFlex N-500	5		0.841			0.725			0.500			0.681			2		0.658		0.616				0.664			0.571			15		0.942			0.396			0.678			0.792			9		0.999			0.477			0.329			0.511	
			0.086			0.106			0.256			0.129					0.149		0.169				0.197			0.160					0.091			0.286			0.253			0.166					0.010			0.264			0.464			0.207	
microPHAZIR	4		0.840			0.460			0.345			0.677			11		0.702		0.317				0.613			0.603			7		0.984			0.700			0.828			0.854			2		0.939			0.095			0.791			0.738	
			0.086			0.121			0.192			0.114					0.155		0.176				0.169			0.160					0.048			0.178			0.337			0.153					0.078			0.291			0.169			0.146	
MicroNIR 1700	12		0.958			0.521			0.607			0.840			7		0.926		0.126				0.566			0.674			7		0.785			0.369			0.242			0.597			9		0.742			0.452			0.416			0.624	
			0.043			0.138			0.136			0.083					0.068		0.299				0.170			0.145					0.162			0.290			0.381			0.230					0.143			0.242			0.215			0.173	
Enterprise	11		1.000			0.598			0.496			0.574			13		0.963		0.128				0.471			0.670			8		0.884			0.519			0.536			0.680			11		0.965			0.602			0.368			0.729	
			0.000			0.107			0.346			0.140					0.051		0.306				0.209			0.150					0.129			0.333			0.310			0.207					0.049			0.230			0.290			0.149	
SCiO	5		0.781			0.277			0.191			0.533			12		0.576		0.017				0.451			0.450			15		0.665			0.219			0.308			0.528			1		0.693			0.272			0.357			0.341	
			0.098			0.168			0.227			0.136					0.164		0.243				0.188			0.182					0.209			0.375			0.268			0.250					0.151			0.216			0.429			0.225	
(**c**)																																																							
		**Hardness**														**Juiciness**														**Fatness**														**Fibrousness**											
		N		R^2^_Train_			R^2^_Valid_			R^2^_Test_			R^2^_Total_			N	R^2^_Train_			R^2^_Valid_				R^2^_Test_			R^2^_Total_			N		R^2^_Train_			R^2^_Valid_			R^2^_Test_			R^2^_Total_			N		R^2^_Train_			R^2^_Valid_			R^2^_Test_			R^2^_Total_
				RMSE			RMSE			RMSE			RMSE				RMSE			RMSE				RMSE			RMSE					RMSE			RMSE			RMSE			RMSE					RMSE			RMSE			RMSE			RMSE
NIRFlex N-500		9		0.996			0.844			0.758			0.926			8	0.903			0.682				0.511			0.700			13		0.853			0.654			0.709			0.821			5		0.864			0.751			0.556			0.781
				0.038			0.219			0.336			0.159				0.127			0.360				0.446			0.246					0.214			0.241			0.191			0.215					0.204			0.313			0.361			0.252
microPHAZIR		6		0.963			0.620			0.898			0.913			10	0.988			0.638				0.811			0.797			11		0.994			0.028			0.757			0.742			15		0.996			0.665			0.734			0.794
				0.121			0.319			0.137			0.168				0.045			0.336				0.404			0.207					0.040			0.522			0.409			0.259					0.040			0.388			0.671			0.302
MicroNIR 1700		14		0.873			0.763			0.882			0.821			15	0.887			0.513				0.526			0.717			11		0.832			0.514			0.610			0.695			7		0.832			0.252			0.797			0.719
				0.190			0.435			0.241			0.250				0.144			0.332				0.391			0.232					0.189			0.413			0.416			0.276					0.216			0.453			0.327			0.282
Enterprise		6		0.968			0.756			0.727			0.851			14	0.957			0.802				0.707			0.850			4		0.998			0.592			0.635			0.737			13		0.998			0.596			0.495			0.804
				0.119			0.346			0.470			0.247				0.084			0.227				0.331			0.171					0.021			0.532			0.514			0.287					0.023			0.510			0.315			0.233
SCiO		2		0.864			0.862			0.760			0.829			2	0.653			0.207				0.802			0.570			9		0.590			0.223			0.200			0.459			14		0.857			0.836			0.430			0.671
				0.170			0.361			0.330			0.237				0.244			0.453				0.237			0.284					0.340			0.397			0.444			0.366					0.208			0.400			0.673			0.350
		**Chewiness**														**Gumminess**														**Heterogeneity**														**Chewing residue**											
		N		R^2^_Train_			R^2^_Valid_			R^2^_Test_			R^2^_Total_			N	R^2^_Train_			R^2^_Valid_				R^2^_Test_			R^2^_Total_			N		R^2^_Train_			R^2^_Valid_			R^2^_Test_			R^2^_Total_			N		R^2^_Train_			R^2^_Valid_			R^2^_Test_			R^2^_Total_
				RMSE			RMSE			RMSE			RMSE				RMSE			RMSE				RMSE			RMSE					RMSE			RMSE			RMSE			RMSE					RMSE			RMSE			RMSE			RMSE
NIRFlex N-500		10		0.999			0.716			0.675			0.748			6	0.882			0.817				0.654			0.757			5		0.809			0.170			0.719			0.682			3		0.938			0.326			0.325			0.614
				0.009			0.347			0.446			0.219				0.092			0.152				0.325			0.159					0.236			0.453			0.354			0.298					0.081			0.297			0.443			0.217
microPHAZIR		10		0.992			0.214			0.533			0.631			4	0.745			0.439				0.603			0.673			13		0.831			0.502			0.513			0.675			15		0.997			0.273			0.635			0.715
				0.034			0.500			0.403			0.250				0.170			0.256				0.216			0.192					0.224			0.551			0.391			0.322					0.021			0.306			0.301			0.167
MicroNIR 1700		11		0.761			0.159			0.629			0.648			3	0.589			0.425				0.411			0.478			4		0.801			0.521			0.737			0.717			10		0.864			0.511			0.313			0.522
				0.197			0.321			0.277			0.232				0.204			0.201				0.386			0.240					0.219			0.470			0.267			0.278					0.105			0.356			0.472			0.245
Enterprise		9		0.963			0.409			0.467			0.702			8	0.776			0.308				0.459			0.470			11		0.949			0.218			0.696			0.665			8		0.966			0.321			0.414			0.715
				0.077			0.266			0.493			0.226				0.166			0.278				0.411			0.237					0.118			0.565			0.613			0.338					0.064			0.324			0.276			0.173
SCiO		11		0.691			0.523			0.443			0.603			9	0.493			0.389				0.349			0.449			15		0.817			0.193			0.703			0.649			15		0.720			0.482			0.574			0.590
				0.212			0.328			0.301			0.247				0.232			0.228				0.254			0.235					0.226			0.596			0.236			0.312					0.160			0.259			0.290			0.202

N: number of neurons in the hidden layer.

**Table 4:** (**a**). Correlation coefficients (R^2^) and root mean square error (RMSE) values for the best ANN obtained from the NIR spectra of the whole slice: Appearance parameters; (**b**). Correlation coefficients (R^2^) and root mean square error (RMSE) values for the best ANN obtained from the NIR spectra of the whole slice: Flavor attributes; (**c**). Correlation coefficients and root mean square error (RMSE) values for the best ANN architecture obtained from the NIR spectra of fat: Texture attributes.

(**a**)																									
	**Veined**										**Fat Color**										**Color Homogeneity**				
	N				R^2^_Train_		R^2^_Valid_	R^2^_Test_		R^2^_Total_	N	R^2^_Train_		R^2^_Valid_			R^2^_Test_		R^2^_Total_		N	R^2^_Train_	R^2^_Valid_	R^2^_Test_	R^2^_Total_
					RMSE		RMSE	RMSE		RMSE		RMSE		RMSE			RMSE		RMSE			RMSE	RMSE	RMSE	RMSE
NIRFlex N-500	12				0.942		0.603	0.847		0.835	13	0.999		0.054			0.947		0.800		15	1.000	0.543	0.746	0.834
					0.267		0.895	0.636		0.481		0.012		0.451			0.198		0.191			0.003	0.467	0.347	0.225
Foss 5000 *	5				0.695		0.662	0.751		0.632	4	0.708		0.284			0.733		0.574		11	0.926	0.193	0.678	0.749
					0.518		0.831	1.024		0.670		0.235		0.459			0.179		0.274			0.145	0.520	0.291	0.261
microPHAZIR	13				0.882		0.434	0.821		0.797	6	0.794		0.458			0.794		0.720		3	0.945	0.650	0.709	0.801
					0.452		0.737	0.583		0.525		0.205		0.340			0.168		0.226			0.112	0.402	0.357	0.228
MicroNIR 1700	1				1.000		0.372	0.708		0.825	9	0.910		0.387			0.697		0.802		9	0.909	0.366	0.632	0.667
					0.000		1.036	0.543		0.453		0.140		0.334			0.164		0.186			0.154	0.432	0.544	0.298
Enterprise	11				0.918		0.394	0.874		0.808	2	0.731		0.587			0.734		0.653		10	1.000	0.615	0.635	0.722
					0.316		0.809	0.607		0.472		0.230		0.450			0.336		0.290			0.004	0.459	0.593	0.290
SCiO	3				0.730		0.716	0.844		0.709	13	0.816		0.830			0.738		0.392		2	0.401	0.223	0.583	0.373
					0.557		0.590	0.688		0.584		0.202		0.152			0.747		0.340			0.412	0.381	0.307	0.393
	**Color intensity**										**Exudate**										**White dots**				
	N				R^2^_Train_		R^2^_Valid_	R^2^_Test_		R^2^_Total_	N	R^2^_Train_		R^2^_Valid_			R^2^_Test_		R^2^_Total_		N	R^2^_Train_	R^2^_Valid_	R^2^_Test_	R^2^_Total_
					RMSE		RMSE	RMSE		RMSE		RMSE		RMSE			RMSE		RMSE			RMSE	RMSE	RMSE	RMSE
NIRFlex N-500	12				0.841		0.639	0.596		0.731	8	0.819		0.762			0.636		0.519		3	0.706	0.144	0.790	0.628
					0.208		0.313	0.464		0.278		0.230		0.310			0.779		0.377			0.423	0.711	0.291	0.462
Foss 5000 *	5				0.750		0.539	0.788		0.634	8	0.818		0.096			0.649		0.636		6	0.625	0.257	0.735	0.423
					0.275		0.361	0.342		0.300		0.216		0.544			0.371		0.313			0.565	0.870	0.458	0.607
microPHAZIR	7				0.868		0.784	0.566		0.758	8	0.917		0.846			0.589		0.407		1	0.974	0.451	0.701	0.735
					0.170		0.436	0.266		0.243		0.150		0.159			0.973		0.402			0.160	0.801	0.682	0.429
MicroNIR 1700	13				0.909		0.696	0.734		0.676	12	0.790		0.304			0.780		0.650		11	0.957	0.158	0.740	0.711
					0.136		0.284	0.579		0.274		0.266		0.456			0.317		0.309			0.170	0.840	0.734	0.455
Enterprise	1				0.917		0.502	0.799		0.782	15	0.720		0.364			0.663		0.511		14	0.687	0.017	0.796	0.537
					0.238		0.335	0.282		0.261		0.291		0.707			0.353		0.391			0.454	0.782	0.509	0.525
SCiO	3				0.726		0.580	0.645		0.690	12	0.670		0.360			0.673		0.429		9	0.877	0.366	0.742	0.683
					0.263		0.288	0.322		0.276		0.293		0.565			0.634		0.410			0.283	0.884	0.440	0.450
(**b**)																									
	**Odor Intensity**										**Cured aroma**										**Pig aroma**				
	N				R^2^_Train_		R^2^_Valid_	R^2^_Test_		R^2^_Total_	N	R^2^_Train_		R^2^_Valid_			R^2^_Test_		R^2^_Total_		N	R^2^_Train_	R^2^_Valid_	R^2^_Test_	R^2^_Total_
					RMSE		RMSE	RMSE		RMSE		RMSE		RMSE			RMSE		RMSE			RMSE	RMSE	RMSE	RMSE
NIRFlex N-500	11				0.997		0.138	0.602		0.752	14	1.000		0.509			0.689		0.900		9	1.000	0.007	0.609	0.715
					0.016		0.322	0.197		0.147		0.000		0.172			0.133		0.084			0.000	0.142	0.088	0.065
Foss 5000 *	10				0.808		0.014	0.771		0.653	10	0.905		0.394			0.683		0.763		10	0.811	0.087	0.773	0.681
					0.132		0.305	0.141		0.171		0.086		0.226			0.135		0.125			0.054	0.117	0.043	0.066
microPHAZIR	10				1.000		0.170	0.679		0.683	12	0.938		0.409			0.834		0.809		6	0.768	0.125	0.600	0.379
					0.002		0.424	0.181		0.179		0.082		0.116			0.203		0.114			0.063	0.142	0.134	0.092
MicroNIR 1700	15				0.918		0.378	0.906		0.689	15	0.552		0.508			0.677		0.412		15	0.772	0.040	0.718	0.642
					0.088		0.287	0.290		0.174		0.166		0.312			0.249		0.208			0.061	0.116	0.081	0.075
Enterprise	5				0.653		0.190	0.746		0.503	12	0.670		0.365			0.705		0.326		8	0.960	0.491	0.632	0.737
					0.173		0.310	0.225		0.207		0.151		0.253			0.369		0.214			0.023	0.100	0.143	0.070
SCiO	4				0.758		0.005	0.623		0.588	14	0.771		0.344			0.582		0.232		2	0.417	0.137	0.665	0.405
					0.138		0.259	0.262		0.184		0.113		0.301			0.457		0.232			0.081	0.107	0.124	0.093
	**Rancid aroma**										**Atypical aroma**										**Flavor intensity**				
	N				R^2^_Train_		R^2^_Valid_	R^2^_Test_		R^2^_Total_	N	R^2^_Train_		R^2^_Valid_			R^2^_Test_		R^2^_Total_		N	R^2^_Train_	R^2^_Valid_	R^2^_Test_	R^2^_Total_
					RMSE		RMSE	RMSE		RMSE		RMSE		RMSE			RMSE		RMSE			RMSE	RMSE	RMSE	RMSE
NIRFlex N-500	4				0.575		0.227	0.625		0.505	14	0.961		0.314			0.643		0.718		8	0.902	0.379	0.540	0.307
					0.139		0.107	0.166		0.139		0.031		0.199			0.103		0.090			0.091	0.291	0.624	0.277
microPHAZIR	10				0.707		0.185	0.710		0.421	6	0.941		0.141			0.557		0.557		9	0.845	0.542	0.761	0.717
					0.100		0.279	0.145		0.148		0.046		0.209			0.197		0.118			0.119	0.198	0.275	0.165
MicroNIR 1700	13				0.562		0.017	0.769		0.245	5	0.995		0.048			0.608		0.616		4	0.810	0.437	0.536	0.645
					0.130		0.279	0.195		0.171		0.016		0.249			0.106		0.106			0.127	0.238	0.306	0.184
Enterprise	14				0.793		0.271	0.643		0.598	13	0.922		0.529			0.654		0.635		2	0.661	0.185	0.595	0.462
					0.098		0.207	0.115		0.123		0.056		0.103			0.225		0.107			0.189	0.288	0.310	0.228
SCiO	14				0.918		0.199	0.747		0.344	10	0.792		0.184			0.618		0.476		2	0.822	0.778	0.750	0.753
					0.057		0.206	0.405		0.182		0.086		0.236			0.229		0.146			0.161	0.145	0.156	0.158
		**Fat flavor intensity**						**Cured flavor**					**Saltiness**								**Sweetness**				
		N		R^2^_Train_	R^2^_Valid_	R^2^_Test_	R^2^_Total_	N	R^2^_Train_	R^2^_Valid_	R^2^_Test_	R^2^_Total_	N		R^2^_Train_	R^2^_Valid_		R^2^_Test_		R^2^_Total_	N	R^2^_Train_	R^2^_Valid_	R^2^_Test_	R^2^_Total_
				RMSE	RMSE	RMSE	RMSE		RMSE	RMSE	RMSE	RMSE			RMSE	RMSE		RMSE		RMSE		RMSE	RMSE	RMSE	RMSE
NIRFlex N-500		11		1.000	0.209	0.638	0.508	9	0.992	0.240	0.524	0.710	15		0.981	0.644		0.745		0.816	15	0.855	0.666	0.707	0.787
				0.000	0.614	0.794	0.389		0.027	0.307	0.334	0.177			0.062	0.275		0.292		0.164		0.095	0.176	0.152	0.120
Foss 5000 *		12		0.758	0.120	0.771	0.611	10	0.973	0.076	0.665	0.427	10		0.972	0.662		0.561		0.787	11	0.823	0.551	0.608	0.381
				0.272	0.497	0.338	0.326		0.062	0.387	0.521	0.257			0.060	0.272		0.301		0.165		0.124	0.187	0.447	0.214
microPHAZIR		2		0.991	0.581	0.653	0.527	14	0.656	0.012	0.635	0.373	9		1.000	0.669		0.600		0.699	4	0.991	0.579	0.773	0.872
				0.142	0.344	0.964	0.414		0.190	0.461	0.293	0.265			0.000	0.212		0.458		0.196		0.025	0.169	0.175	0.096
MicroNIR 1700		4		0.999	0.477	0.576	0.727	14	0.741	0.263	0.560	0.386	5		0.646	0.158		0.605		0.510	15	1.000	0.500	0.639	0.851
				0.052	0.370	0.675	0.301		0.182	0.215	0.430	0.240			0.203	0.388		0.272		0.250		0.002	0.206	0.142	0.097
Enterprise		11		0.897	0.026	0.731	0.430	11	0.967	0.573	0.713	0.826	9		0.940	0.808		0.825		0.867	5	0.747	0.303	0.669	0.587
				0.167	0.974	0.513	0.448		0.056	0.272	0.189	0.137			0.086	0.166		0.223		0.130		0.122	0.288	0.158	0.163
SCiO		15		0.601	0.604	0.589	0.569	14	0.794	0.055	0.855	0.214	1		0.982	0.764		0.587		0.877	13	0.739	0.573	0.618	0.670
				0.329	0.340	0.405	0.343		0.133	0.383	0.540	0.280			0.189	0.231		0.299		0.216		0.125	0.164	0.205	0.146
		**Sourness**						**Rancidity**					**Aftertaste**								**Atypical flavor**				
		N		R^2^_Train_	R^2^_Valid_	R^2^_Test_	R^2^_Total_	N	R^2^_Train_	R^2^_Valid_	R^2^_Test_	R^2^_Total_	N		R^2^_Train_	R^2^_Valid_		R^2^_Test_		R^2^_Total_	N	R^2^_Train_	R^2^_Valid_	R^2^_Test_	R^2^_Total_
				RMSE	RMSE	RMSE	RMSE		RMSE	RMSE	RMSE	RMSE			RMSE	RMSE		RMSE		RMSE		RMSE	RMSE	RMSE	RMSE
NIRFlex N-500		3		0.914	0.095	0.507	0.726	8	0.809	0.228	0.565	0.599	12		0.789	0.049		0.599		0.598	8	0.800	0.398	0.640	0.643
				0.061	0.176	0.154	0.104		0.122	0.272	0.127	0.155			0.161	0.363		0.316		0.230		0.127	0.258	0.229	0.171
		10		0.784	0.177	0.686	0.614	9	0.769	0.191	0.697	0.220	13		0.889	0.630		0.682		0.743	7	0.939	0.561	0.787	0.378
				0.104	0.190	0.136	0.126		0.117	0.381	0.340	0.221			0.111	0.353		0.244		0.190		0.073	0.200	0.571	0.242
microPHAZIR		11		1.000	0.145	0.699	0.781	15	0.909	0.593	0.607	0.422	5		0.926	0.381		0.627		0.740	7	0.849	0.449	0.629	0.697
				0.005	0.211	0.134	0.097		0.084	0.129	0.465	0.200			0.098	0.234		0.382		0.192		0.113	0.199	0.243	0.154
MicroNIR 1700		5		0.999	0.499	0.602	0.827	5	0.655	0.472	0.772	0.302	2		0.995	0.524		0.572		0.737	1	0.938	0.563	0.721	0.605
				0.005	0.147	0.173	0.088		0.144	0.194	0.405	0.212			0.031	0.292		0.471		0.216		0.111	0.159	0.401	0.191
Enterprise		13		1.000	0.100	0.606	0.386	13	0.915	0.137	0.672	0.169	4		0.814	0.690		0.779		0.676	6	0.880	0.010	0.741	0.319
				0.003	0.212	0.443	0.190		0.078	0.311	0.620	0.276			0.152	0.279		0.365		0.219		0.105	0.312	0.506	0.247
SCiO		8		0.395	0.014	0.661	0.257	14	0.791	0.489	0.606	0.473	13		0.748	0.187		0.646		0.613	7	0.653	0.173	0.688	0.154
				0.162	0.201	0.179	0.171		0.114	0.258	0.283	0.176			0.175	0.343		0.318		0.233		0.148	0.264	0.580	0.276
(**c**)																									
		**Hardness**						**Juiciness**					**Fatness**								**Fibrousness**				
		N	R^2^_Train_		R^2^_Valid_	R^2^_Test_	R^2^_Total_	N	R^2^_Train_	R^2^_Valid_	R^2^_Test_	R^2^_Total_	N		R^2^_Train_	R^2^_Valid_		R^2^_Test_		R^2^_Total_	N	R^2^_Train_	R^2^_Valid_	R^2^_Test_	R^2^_Total_
			RMSE		RMSE	RMSE	RMSE		RMSE	RMSE	RMSE	RMSE			RMSE	RMSE		RMSE		RMSE		RMSE	RMSE	RMSE	RMSE
NIRFlex N-500		12	1.000		0.725	0.860	0.905	5	0.999	0.549	0.907	0.919	15		0.971	0.004		0.713		0.596	12	0.982	0.561	0.793	0.830
			0.000		0.255	0.402	0.184		0.011	0.230	0.235	0.128			0.093	0.867		0.325		0.367		0.086	0.365	0.434	0.231
Foss 5000 *		8	0.997		0.595	0.686	0.426	2	0.672	0.578	0.663	0.610	11		0.982	0.137		0.728		0.805	3	0.849	0.345	0.690	0.705
			0.037		0.508	1.066	0.458		0.230	0.408	0.289	0.273			0.070	0.490		0.285		0.227		0.199	0.353	0.484	0.286
microPHAZIR		2	0.935		0.540	0.914	0.780	13	0.980	0.546	0.785	0.822	1		0.961	0.684		0.607		0.568	8	0.918	0.687	0.784	0.823
			0.135		0.565	0.285	0.270		0.059	0.339	0.308	0.184			0.169	0.343		0.720		0.340		0.138	0.418	0.250	0.221
MicroNIR 1700		10	0.955		0.773	0.868	0.853	2	0.875	0.631	0.875	0.794	2		0.741	0.248		0.683		0.627	15	0.795	0.681	0.804	0.711
			0.116		0.234	0.491	0.232		0.174	0.327	0.198	0.208			0.234	0.508		0.328		0.305		0.244	0.512	0.132	0.289
Enterprise		1	0.986		0.594	0.733	0.852	1	0.856	0.127	0.718	0.654	2		0.558	0.630		0.755		0.239	9	0.806	0.412	0.627	0.662
			0.113		0.387	0.335	0.220		0.218	0.390	0.485	0.302			0.321	0.367		0.818		0.439		0.233	0.550	0.337	0.317
SCiO		7	0.791		0.615	0.907	0.760	4	0.969	0.469	0.813	0.848	11		0.665	0.287		0.706		0.525	3	0.834	0.569	0.784	0.756
			0.299		0.335	0.410	0.324		0.072	0.349	0.206	0.168			0.279	0.392		0.542		0.349		0.189	0.368	0.380	0.259
		**Chewiness**						**Gumminess**					**Heterogeneity**								**Chewing residue**				
		N	R^2^_Train_		R^2^_Valid_	R^2^_Test_	R^2^_Total_	N	R^2^_Train_	R^2^_Valid_	R^2^_Test_	R^2^_Total_	N		R^2^_Train_	R^2^_Valid_		R^2^_Test_		R^2^_Total_	N	R^2^_Train_	R^2^_Valid_	R^2^_Test_	R^2^_Total_
			RMSE		RMSE	RMSE	RMSE		RMSE	RMSE	RMSE	RMSE			RMSE	RMSE		RMSE		RMSE		RMSE	RMSE	RMSE	RMSE
NIRFlex N-500		15	0.975		0.683	0.761	0.870	13	0.933	0.257	0.609	0.647	15		0.978	0.710		0.813		0.857	11	0.997	0.200	0.804	0.795
			0.061		0.272	0.215	0.143		0.089	0.252	0.436	0.209			0.082	0.192		0.500		0.219		0.018	0.295	0.286	0.160
Foss 5000 *		14	0.820		0.091	0.691	0.584	10	0.941	0.043	0.622	0.587	14		1.000	0.349		0.707		0.814	14	0.916	0.004	0.689	0.293
			0.181		0.397	0.351	0.255		0.073	0.454	0.254	0.211			0.000	0.383		0.559		0.263		0.094	0.435	0.531	0.277
microPHAZIR		15	0.848		0.053	0.532	0.540	7	0.904	0.306	0.698	0.455	9		0.839	0.246		0.687		0.712	8	0.714	0.043	0.699	0.304
			0.161		0.518	0.305	0.269		0.106	0.262	0.503	0.237			0.242	0.365		0.413		0.294		0.176	0.391	0.430	0.269
MicroNIR 1700		12	0.958		0.529	0.724	0.782	12	0.704	0.049	0.820	0.558	14		0.874	0.406		0.769		0.722	2	0.862	0.370	0.813	0.708
			0.074		0.359	0.287	0.189		0.183	0.369	0.157	0.218			0.199	0.487		0.325		0.281		0.131	0.269	0.254	0.180
Enterprise		9	0.927		0.502	0.822	0.341	11	0.886	0.609	0.637	0.337	11		0.911	0.108		0.732		0.710	15	0.816	0.105	0.574	0.522
			0.114		0.422	0.783	0.358		0.109	0.253	0.652	0.286			0.157	0.570		0.290		0.280		0.137	0.409	0.237	0.216
SCiO		7	0.907		0.338	0.901	0.711	1	0.662	0.011	0.738	0.605	15		0.710	0.509		0.759		0.649	10	0.996	0.572	0.558	0.617
			0.114		0.401	0.378	0.234		0.225	0.194	0.160	0.212			0.303	0.364		0.279		0.309		0.020	0.284	0.503	0.224

N: number of neurons in the hidden layer. * Foss 5000 spectra were recorded at three different points (5 cm × 5 cm, including fat and lean meat), and their average was the whole slice spectra. The whole slice spectra for the rest of the devices were the mean value of 6 spectra of the lean meat and 4 spectra of the fat.

**Table 5:** Number of parameters predicted depending on the R^2^_Total_ for each recording site and device.

		Lean Meat				Fat				Whole Slice			
Device	R^2^_Total_	Appearance (6)	Flavor (14)	Texture (8)	**Total** **(28)**	Appearance (6)	Flavor (14)	Texture (8)	**Total** **(28)**	Appearance (6)	Flavor (14)	Texture (8)	**Total** **(28)**
NIRFlex N-500	>0.5	6	13	7	26	6	13	8	27	6	13	8	27
	>0.6	5	12	5	22	5	10	8	23	5	9	7	21
	>0.7	4	6	4	14	4	6	6	16	4	8	6	18
	>0.8	2	1	3	6	2	2	2	6	3	2	5	10
Foss 5000	>0.5									5	9	6	20
	>0.6									3	8	4	15
	>0.7									1	4	3	8
	>0.8									0	0	2	2
microPHAZIR	>0.5	5	12	4	21	6	13	8	27	5	10	6	21
	>0.6	4	10	3	17	6	10	8	24	5	8	4	15
	>0.7	2	6	1	9	4	4	5	13	5	4	4	13
	>0.8	2	1	0	3	2	1	1	4	1	2	2	5
MicroNIR 1700	>0.5	6	12	7	25	6	12	7	25	6	10	8	25
	>0.6	5	7	7	19	6	10	6	22	6	8	7	21
	>0.7	3	4	4	11	3	4	3	10	3	4	2	9
	>0.8	2	1	2	5	0	2	1	5	2	2	0	4
Enterprise	>0.5	6	12	7	25	6	13	7	26	6	7	5	18
	>0.6	5	8	6	19	6	11	7	24	4	8	4	13
	>0.7	1	4	1	6	4	3	6	13	3	4	2	9
	>0.8	1	1	1	3	1	0	3	4	1	2	1	4
SCiO	>0.5	6	10	7	23	6	8	6	20	3	6	8	17
	>0.6	2	6	6	14	4	2	4	10	3	2	7	12
	>0.7	2	4	4	10	0	1	1	2	0	1	4	6
	>0.8	0	1	2	3	0	1	1	2	0	1	0	1

## Data Availability

The data presented in this study are openly available in the Gredos repository: http://hdl.handle.net/10366/168068 (accessed on 21 January 2026).

## Supplementary Materials

The following supporting information can be downloaded at https://www.mdpi.com/article/10.3390/foods15030436/s1: Table S1. Sensory parameters evaluated by the assessors and the definition of sensory parameters; Table S2. Technical characteristics of the devices used in the study; Table S3: Ratio performance deviation (RPD) values for the best ANN architecture.
