# Supplementary material for: The Influence of the Site of Recording and Benchtop and Portable NIRS Equipment on Predicting the Sensory Properties of Iberian Ham
Source: Foods. 2026 Jan 24;15(3):436. doi: 10.3390/foods15030436 (PMC12896908; doi:10.3390/foods15030436)
Supplement: Supplementary file 1 [file foods-15-00436-s001.zip › Table S1.pdf]

Table S1. Sensory parameters evaluated by the assessors and the definition of sensory parameters

Parameter	Description	Score criteria	
Visual		1	9
Veined	Amount of intramuscular fat	Absence of intramuscular fat	Large amount of intramuscular fat
Fat color	Color shade of the intramuscular fat	Yellow	White
Color homogeneity	Presence or absence of the various shades	Inhomogeneous	Homogeneous
Color intensity	Color intensity of the item	Pink	Red
Exudate	Shine from the separation of fat on the surface	Absence of exudate	High intensity of exudate
White dots	Presence of white dots owing to the precipitation of tyrosine	Absence of white dots	Large number of white dots
Flavor			
Odor	Intensity of odor before eating	Low intensity of odor	High intensity of odor
Cured aroma	Odor of cured meat	Low intensity of cured aroma	High intensity of cured aroma
Pig aroma	Odor of abattoir or recently slaughtered pig	Absence of pig aroma	High intensity of pig aroma
Rancidity aroma	Intensity of rancid odor	Low intensity of rancid odor	High intensity of rancid odor
Atypical aroma	Presence of strange odors uncharacteristic of ham	Absence of atypical aroma	High intensity of atypical aroma
Flavor intensity	Sensation of flavors once the product has been placed in the mouth	Low intensity of flavor	High intensity of flavor
Fat flavor intensity	Flavor intensity of the fat fraction	Low intensity of fat flavor	High intensity of fat flavor
Cured flavor	Intensity of cured flavor	Low intensity of cured flavor	High intensity of cured flavor
Saltiness	Intensity of salty taste	Low intensity of saltiness	High intensity of saltiness
Sweetness	Intensity of sweet taste	Low intensity of sweetness	High intensity of sweetness
Sourness	Intensity of acid taste	Low intensity of sourness	High intensity of sourness
Rancidity	Intensity of rancid flavor	Low intensity of rancid flavor	High intensity of rancid flavor
Aftertaste	Persistence of the taste after having eaten the product	Low intensity of aftertaste	High intensity of aftertaste
Atypical flavor	Presence of strange odors uncharacteristic of ham	Absence of atypical flavor	High intensity of atypical flavor
Texture			
Hardness	Firmness perception during chewing	Low intensity of firmness	High intensity of firmness
Juiciness	Impression of juiciness during chewing	Low intensity of juiciness	High intensity of rancid odor
Fatness	Appearance of a fatty sensation when chewing the product	Low intensity of fatness	High intensity of fatness
Fibrousness	Perception of fibers during chewing	Low number of fibers	High number of fibers
Chewiness	No. of bites necessary before the item is swallowed	Few bites	Many bites

Gumminess	Tendency to form a ball when the product is chewed	Low intensity of gumminess	High intensity of gumminess
Heterogeneity	Presence or absence of different textures in the item on chewing it	Homogeneity	Lack of homogeneity
Chewing Residue	If remains of the product stay in the mouth once we have swallowed it	Little or no residue	Large amount of residue
