# Supplementary material for: The Influence of the Site of Recording and Benchtop and Portable NIRS Equipment on Predicting the Sensory Properties of Iberian Ham
Source: Foods. 2026 Jan 24;15(3):436. doi: 10.3390/foods15030436 (PMC12896908; doi:10.3390/foods15030436)
Supplement: Supplementary file 1 [file foods-15-00436-s001.zip › Table S2.pdf]

Table S2. Technical characteristics of the devices used in the study

Spectrometer (Vendor)	Key components			Spectral Region		Resolution	Conectivity	Dimensions	Weight
	Source	Wavelength Selector	Detector	[nm]	[cm^{-1}]	[nm]	Cata Transfer	[cm]	[g]
NIRSystem 5000 (Foss)	Tungsten-halogen lamp		Lead sulphide (PbS) photodetector	1100-2000	9090-5000	2	Wire	15 x 21 x 11	19000
NIRFlex N-500 (Büchi)	Tungsten halogen (duplicated)	Polarization interferometer (TiO_2 wedges)	InGaAs (single-element, thermoelectric cooling)	1000-2500	10000-4000	~2	Ethernet	45 × 35 × 25	15000
MircroNIR 1700 ES (VIAVI)	Tungsten halogen (duplicated)	LVF	InGaAs (array; 128 elements)	908–1676	11013–5967	12.5 (1000) 25 (2000)	USB-Control and power delivery	5.0 × 4.6 (Ø)	58
Enterprise Scanner NIR-S-G1 (Tellspec)	Tungsten halogen (duplicated)	Stationary dispersive grating and MEMS DMD	InGaAs (single-element)	900–1700	11111–5882	10	Bluetooth (Cloud service)	8.2 × 6.3 × 4.0	136
SCiO (Consumer Physics)	LED	Bandpass filter	Si photodiode (CMOS) array (12 elements)	740–1070	13514–9346	Not disclosed	Bluetooth (Cloud service)	6.8 × 3.9 × 1.5	35
microPHAZIR (ThermoScientific)	Tungsten halogen	MEMS Hadamard mask	InGaAs (single element)	1596-2395	6267-4173	8	Wireless with battery and internal memory	25.4 x 29.2 x 15.2	1250

Abbreviations: CMOS—Complementary Metal–Oxide–Semiconductor; DMD—Digital Micromirror Device; InGaAs—Indium Galium Arsenide; LED—Light Emitting Diode; LVF—Linear Variable Filter; MEMS—Micro-Electro-Mechanical System; USB—Universal Serial Bus; Si—Silicon; NIR—near-infrared.
