# Supplementary material for: The Influence of the Site of Recording and Benchtop and Portable NIRS Equipment on Predicting the Sensory Properties of Iberian Ham
Source: Foods. 2026 Jan 24;15(3):436. doi: 10.3390/foods15030436 (PMC12896908; doi:10.3390/foods15030436)
Supplement: Supplementary file 1 [file foods-15-00436-s001.zip › Table S3.pdf]

Table S3. Ratio Performance Deviation (RPD) values for the best ANN architecture

S3a. Ratio Performance Deviation (RPD) values for the best ANN architecture obtained from the NIR spectra of lean meat.

| Appearance | | | | | | | | |
|---|---|---|---|---|---|---|---|---|
| | Veined | Fat color | Color homogeneity | Color intensity | Exudate | White dots | | |
| NIRFlex N500 | 2.31 | 2.55 | 1.94 | 2.12 | 1.48 | 1.53 | | |
| MicroPHAZIR | 2.17 | 1.70 | 2.23 | 1.61 | 1.31 | 1.45 | | |
| MicroNIR | 2.43 | 1.19 | 3.00 | 1.78 | 1.40 | 1.61 | | |
| Enterprise | 2.34 | 1.64 | 1.48 | 1.61 | 1.50 | 1.54 | | |
| SCiO | 1.83 | 1.47 | 1.55 | 1.40 | 1.34 | 2.04 | | |
| Flavour | | | | | | | | |
| | Odor Intensity | Cured aroma | Pig aroma | Rancid aroma | Atypical aroma | Flavor intensity | | |
| NIRFlex N500 | 1.51 | 1.52 | 1.69 | 2.07 | 1.55 | 1.91 | | |
| MicroPHAZIR | 1.59 | 1.76 | 2.02 | 1.50 | 1.53 | 1.92 | | |
| MicroNIR | 1.29 | 1.30 | 1.80 | 1.12 | 1.69 | 1.99 | | |
| Enterprise | 1.54 | 1.70 | 1.83 | 1.57 | 1.49 | 1.92 | | |
| SCiO | 1.52 | 1.78 | 2.60 | 1.36 | 1.37 | 1.89 | | |
| | Fat flavor intensity | Cured flavour | Saltines | Sweetness | Sourness | Rancidity | Aftertaste | Atypical flavor |
| NIRFlex N500 | 1.36 | 1.59 | 1.72 | 1.68 | 1.74 | 2.35 | 1.22 | 1.86 |
| MicroPHAZIR | 2.15 | 1.28 | 1.56 | 2.80 | 1.87 | 1.32 | 1.29 | 1.44 |
| MicroNIR | 1.62 | 1.28 | 1.94 | 2.25 | 1.44 | 0.98 | 1.36 | 1.44 |
| Enterprise | 1.47 | 1.72 | 1.77 | 1.62 | 1.41 | 1.20 | 2.49 | 1.22 |
| SCiO | 1.64 | 1.50 | 1.54 | 1.80 | 1.27 | 1.23 | 1.51 | 1.37 |
| Texture | | | | | | | | |
| | Hardness | Juiciness | Fatness | Fibrousness | Chewiness | Gumminess | Heterogeneity | Chewing residue |
| NIRFlex N500 | 2.55 | 2.96 | 1.42 | 2.43 | 1.31 | 1.08 | 2.04 | 1.39 |
| MicroPHAZIR | 1.71 | 1.89 | 1.48 | 1.54 | 1.23 | 1.29 | 1.22 | 1.26 |
| MicroNIR | 2.68 | 2.49 | 1.64 | 1.73 | 2.13 | 1.55 | 1.54 | 1.30 |
| Enterprise | 1.39 | 1.46 | 1.06 | 2.43 | 1.75 | 1.78 | 1.71 | 1.01 |
| SCiO | 2.18 | 1.83 | 1.83 | 2.38 | 1.65 | 1.45 | 1.66 | 1.21 |

S3b. Ratio Performance Deviation (RPD) values for the best ANN architecture obtained from the NIR spectra of fat.

Appearance

| | Veined | Fat color | Color homogeneity | Color intensity | Exudate | White dots |
|---|---|---|---|---|---|---|
| NIRFlex N500 | 1.94 | 1.67 | 1.30 | 2.13 | 1.42 | 2.37 |
| MicroPHAZIR | 2.31 | 2.45 | 0.77 | 1.68 | 1.66 | 2.55 |
| MicroNIR | 1.71 | 1.87 | 1.19 | 1.58 | 1.56 | 1.41 |
| Enterprise | 1.93 | 2.11 | 0.80 | 1.80 | 2.34 | 1.50 |
| SCiO | 1.62 | 1.50 | 1.18 | 1.47 | 1.48 | 1.78 |

Flavour

| | Odor Intensity | Cured aroma | Pig aroma | Rancid aroma | Atypical aroma | Flavor intensity | | |
|---|---|---|---|---|---|---|---|---|
| NIRFlex N500 | 1.47 | 1.75 | 1.60 | 1.03 | 2.63 | 1.34 | | |
| MicroPHAZIR | 1.99 | 1.59 | 1.33 | 1.09 | 1.69 | 2.10 | | |
| MicroNIR | 1.53 | 1.37 | 1.77 | 1.50 | 1.61 | 1.20 | | |
| Enterprise | 1.49 | 1.28 | 2.16 | 1.61 | 1.76 | 1.65 | | |
| SCiO | 1.39 | 1.19 | 1.60 | 1.51 | 2.28 | 1.26 | | |
| | Fat flavor intensity | Cured flavour | Saltines | Sweetness | Sourness | Rancidity | Aftertaste | Atypical flavor |
| NIRFlex N500 | 1.98 | 2.38 | 1.77 | 1.93 | 1.54 | 1.53 | 2.19 | 1.33 |
| MicroPHAZIR | 1.45 | 1.74 | 1.55 | 1.45 | 1.74 | 1.53 | 2.38 | 1.89 |
| MicroNIR | 1.80 | 1.64 | 1.58 | 2.55 | 2.39 | 1.69 | 1.58 | 1.59 |
| Enterprise | 1.68 | 1.66 | 1.62 | 1.54 | 1.42 | 1.63 | 1.76 | 1.85 |
| SCiO | 1.29 | 1.43 | 1.37 | 1.36 | 1.46 | 1.34 | 1.46 | 1.23 |

Texture

| | Hardness | Juiciness | Fatness | Fibrousness | Chewiness | Gumminess | Heterogeneity | Chewing residue |
|---|---|---|---|---|---|---|---|---|
| NIRFlex N500 | 3.59 | 1.77 | 2.34 | 2.08 | 1.80 | 2.00 | 1.76 | 1.45 |
| MicroPHAZIR | 3.39 | 2.11 | 1.94 | 1.73 | 1.57 | 1.66 | 1.63 | 1.88 |
| MicroNIR | 2.28 | 1.88 | 1.82 | 1.86 | 1.69 | 1.32 | 1.89 | 1.28 |
| Enterprise | 2.31 | 2.55 | 1.75 | 2.25 | 1.74 | 1.34 | 1.55 | 1.81 |
| SCiO | 2.41 | 1.53 | 1.37 | 1.50 | 1.59 | 1.35 | 1.68 | 1.55 |

S3c. Ratio Performance Deviation (RPD) values for the best ANN architecture obtained from the NIR spectra of whole slice.

Appearance

| | Veined | Fat color | Color homogeneity | Color intensity | Exudate | White dots |
|---|---|---|---|---|---|---|
| NIRFlex N500 | 2.26 | 2.20 | 2.19 | 1.71 | 1.34 | 1.65 |
| Foss 5000 | 1.62 | 1.54 | 1.88 | 1.59 | 1.61 | 1.26 |
| MicroPHAZIR | 2.07 | 1.86 | 2.16 | 1.96 | 1.26 | 1.78 |
| MicroNIR | 2.40 | 2.26 | 1.65 | 1.74 | 1.64 | 1.67 |
| Enterprise | 2.30 | 1.45 | 1.70 | 1.82 | 1.29 | 1.45 |
| SCiO | 1.86 | 1.24 | 1.25 | 1.72 | 1.23 | 1.69 |

Flavour

| | Odor Intensity | Cured aroma | Pig aroma | Rancid aroma | Atypical aroma | Flavor intensity |
|---|---|---|---|---|---|---|
| NIRFlex N500 | 1.93 | 3.08 | 1.80 | 1.40 | 1.90 | 1.08 |
| Foss 5000 | 1.66 | 2.07 | 1.77 | 1.31 | 1.45 | 1.81 |
| MicroPHAZIR | 1.58 | 2.27 | 1.27 | 1.14 | 1.61 | 1.62 |
| MicroNIR | 1.63 | 1.24 | 1.56 | 1.58 | 1.60 | 1.31 |
| Enterprise | 1.37 | 1.21 | 1.67 | 1.07 | 1.17 | 1.89 |
| SCiO | 1.54 | 1.11 | 1.26 | 1.43 | 1.18 | 1.13 |

| | Fat flavor intensity | Cured flavour | Saltines | Sweetness | Sourness | Rancidity | Aftertaste | Atypical flavor |
|---|---|---|---|---|---|---|---|---|
| NIRFlex N500 | 1.35 | 1.70 | 2.15 | 2.10 | 1.90 | 1.58 | 1.58 | 1.61 |
| Foss 5000 | 1.61 | 1.17 | 2.14 | 1.18 | 1.57 | 1.11 | 1.92 | 1.14 |
| MicroPHAZIR | 1.27 | 1.13 | 1.80 | 2.63 | 2.04 | 1.22 | 1.90 | 1.79 |
| MicroNIR | 1.75 | 1.25 | 1.41 | 2.60 | 2.25 | 1.15 | 1.69 | 1.44 |
| Enterprise | 1.17 | 2.19 | 2.72 | 1.55 | 1.04 | 0.89 | 1.66 | 1.12 |
| SCiO | 1.53 | 1.07 | 1.64 | 1.73 | 1.16 | 1.39 | 1.56 | 1.00 |

Texture

| | Hardness | Juiciness | Fatness | Fibrousness | Chewiness | Gumminess | Heterogeneity | Chewing residue |
|---|---|---|---|---|---|---|---|---|
| NIRFlex N500 | 3.10 | 3.40 | 1.37 | 2.27 | 2.75 | 1.52 | 2.39 | 1.96 |
| Foss 5000 | 1.25 | 1.60 | 2.22 | 1.83 | 1.54 | 1.51 | 1.99 | 1.13 |
| MicroPHAZIR | 2.11 | 2.37 | 1.48 | 2.37 | 1.46 | 1.34 | 1.78 | 1.17 |
| MicroNIR | 2.46 | 2.10 | 1.65 | 1.81 | 2.08 | 1.46 | 1.87 | 1.74 |
| Enterprise | 2.59 | 1.44 | 1.15 | 1.65 | 1.10 | 1.11 | 1.87 | 1.45 |
| SCiO | 1.76 | 2.59 | 1.44 | 2.02 | 1.68 | 1.50 | 1.70 | 1.40 |
